# Do Local Sex Ratios Approximate Subjective Partner Markets? Evidence from the German Family Panel

**DOI:** 10.1007/s12110-021-09397-6

**Published:** 2021-06-19

**Authors:** Andreas Filser, Richard Preetz

**Affiliations:** Institute for Social Sciences, University of Oldenburg, Ammerlaender Heerstr. 114-118, D-26129 Oldenburg, Germany

**Keywords:** Sex ratio, Partner market, Subjective experience, Relationship formation

## Abstract

Sex ratios have widely been recognized as an important link between demographic contexts and behavior because changes in the ratio shift sex-specific bargaining power in the partner market. Implicitly, the literature considers individual partner market experiences to be a function of local sex ratios. However, empirical evidence on the correspondence between subjective partner availability and local sex ratios is lacking so far. In this paper, we analyzed how closely a set of different local sex ratio measures correlates with subjective partner market experiences. Linking a longitudinal German survey to population data for different entities (states, counties, municipalities), we used multilevel logistic regression models to explore associations between singles’ subjective partner market experiences and various operationalizations of local sex ratios. Results suggest that local sex ratios correlated only weakly with subjective partner market experiences. Adult sex ratios based on broad age brackets, including those for lower-level entities, did not significantly predict whether individuals predominantly met individuals of their own sex. More fine-grained, age-specific sex ratios prove to be better predictors of subjective partner market experiences, in particular when age hypergamy patterns were incorporated. Nevertheless, the respective associations were only significant for selected measures. In a complementary analysis, we illustrate the validity of the subjective indicator as a predictor of relationship formation. In sum, our results suggest that subjective partner availability is not adequately represented by the broad adult sex ratio measures that are frequently used in the literature. Future research should be careful not to equate local sex ratios and conscious partner market experiences.

**Supplementary Information:**

The online version contains supplementary material available at 10.1007/s12110-021-09397-6.

Imbalanced proportions of men and women (i.e., skewed sex ratios) have been widely recognized to be a demographic determinant of human behavior. A growing body of literature has pointed out that imbalanced local sex ratios correlate with social consequences, including investment decisions, the participation of females in the labor force, sexual behavior and the spread of HIV, as well as the incidence of violence and aggression (Bien et al., 2013; Den Boer & Hudson, 2004; Diamond-Smith & Rudolph, 2018; Durante et al., 2012; Edlund et al., 2013; Feingold, 2011; Filser et al., 2020; Griskevicius et al., 2012; Merli & Hertog, 2010; Schacht et al., 2014, 2016; Schmitt, 2005; Schnettler & Filser, 2015; Trent & South, 1988, 2012; Trent et al., 2015). The consequences of scarcities of either sex on family formation have received special attention in the literature. Specifically, there is evidence that local sex ratio biases are associated with the age and proportion of individuals marrying and with reproductive outcomes, as well as union formation and stability (Bauer & Kneip, 2013; Cohen & Pepin, 2018; Filser & Schnettler, 2019; Harknett, 2008; Kruger et al., 2010; McLaughlin et al., 1993; Nozaki & Matsuura, 2010; Pollet & Nettle, 2008; Schacht & Kramer, 2016; Schacht & Smith, 2017; South et al., 2001; Trent & South, 1989, 2011; Uggla & Mace, 2016, 2017; Warner et al., 2011). Authors have taken a variety of theoretical perspectives to explain these findings (see Stone, 2015 for a review). All approaches share the underlying premise that the local number of males and females constitute a partner market, which operates by supply-and-demand dynamics (Fossett & Kiecolt, 1991; Guttentag & Secord, 1983; Schacht et al., 2017; Schacht & Kramer, 2016). Specifically, scholars have argued that when one sex outnumbers the other, the rarer sex holds more bargaining power and thus can leverage its scarcity both on the partner market and within relationships (Guttentag & Secord, 1983; Schacht & Kramer, 2016; Uggla & Mace, 2017). Consequently, local sex ratios have been predicted to shift the incentives and opportunities to pursue different partner market strategies, which elicit responses in individual mating behavior (Fossett & Kiecolt, 1991; Guttentag & Secord, 1983; Kokko & Jennions, 2008; Pedersen, 1991; Schacht et al., 2017; Stone, 2015).

Yet, despite the growing literature, the understanding of the nexus between population-level sex ratio skews and individual behavior remains limited. Analytically, the literature postulates a contextual hypothesis linking contextual-level sex ratios with individual behavior on the micro level (Coleman, 1986; Pollet et al., 2017). When studying contextual hypotheses suggesting behavioral adaptations to the social environment, a crucial question is how closely the contextual data reflect individual experiences (Gilbert et al., 2016; Iversen, 1991; Nettle et al., 2012). However, the sex ratio literature so far has been inexplicit regarding the relation of subjective partner market experiences to contextual local sex ratios. This particularly concerns age and geographic boundaries to determine which individuals should be included when calculating local sex ratios. Sociological partner market research has emphasized that life in modern societies is structured into “social foci,” such as workplaces, voluntary organizations, or hangouts (Feld, 1981; Rapp et al., 2015). This insight is not necessarily at odds with the purported effect of local sex ratios because what matters is that the local proportion of men and women influences an individual’s partner pool (Guttentag & Secord, 1983; Obersneider et al., 2018; South et al., 2001). On average, sex ratios in social foci could still be male- or female-skewed, depending on the local distribution of men and women.

Given the emphasis on bargaining power dynamics in the sex ratio literature, the link between local sex ratios and subjective partner market experiences deserves special attention. From an ultimate perspective, it makes adaptive sense for individuals to adjust their behavior to their local ecology, including the sex ratio (Kokko & Jennions, 2008; Pedersen, 1991; Schacht & Kramer, 2016; Stone, 2015). However, this does not answer the question of what the proximate mechanisms are: how do individuals adapt their behavior to the local sex ratio? Prior research on behavioral adaptations to ecological contexts has demonstrated that subjective experiences of the surrounding community provide crucial guidelines for individual decision-making and behavior (Gintis, 2006; Kroneberg & Kalter, 2012; Nettle et al., 2012). Consequently, behavioral adaptations should be particularly pronounced when individuals realize their (un-)favorable position on the partner market. This of course does not preclude alternative, unconscious pathways. For instance, hormonal variation has been cited as an alternative link between local sex ratios and behavior in the literature (Barber, 2009; Maner & McNulty, 2013; Schacht & Kramer, 2016). Unconscious, endocrinal or other processes might either interact with conscious sex ratio experiences or constitute independent pathways. Nevertheless, subjective evaluations of local ecologies have been found to predict attitudes toward violence and mating (Copping & Campbell, 2015). This suggests that subjective experiences of contextual characteristics are an important pathway through which contexts influence individual behavior. Yet, whether or not these subjective evaluations are accurate has thus far remained largely untested (Gilbert et al., 2016). As yet, very little is understood about how humans process contextual sex ratios or which population boundaries individuals rely on (Dillon et al., 2017; Maner & Ackerman, 2020). This raises the question of how closely local sex ratios capture subjective experiences and assessments of partner markets.

The literature has built on the implicit assumption that microlevel subjective partner market experiences are, at least on average, represented by contextual local sex ratios. However, there has been little empirical evidence to support the assumption that subjective partner markets correspond to local sex ratio imbalances. To the contrary, one recent study reported that neighborhood sex ratios in Belfast are uncorrelated with perceptions of respondents in a street survey (Gilbert et al., 2016). Nonetheless, although individuals may be unable to evaluate the sex ratios of specific neighborhoods, the subjective experience of partner markets may correlate with regional, county, or municipality sex ratios because they approximate partner market boundaries more accurately. Most previous studies have analyzed the consequences of sex ratio variation on a larger geographic scale, using data spanning municipalities, counties, regions, or even countries. This appears appropriate because studies have consistently shown the regional nature of partner markets, including online dating markets (Bruch & Newman, 2019; Haandrikman et al., 2008). At the same time, these studies suggest that partner markets are generally larger than neighborhoods. Therefore, microlevel individual partner market experiences might be better captured by sex ratios for larger areas than neighborhoods. However, to our best knowledge, no study has explored the correlation between subjective partner market experiences and sex ratios for larger populations.

In this study, we analyzed how contextual local sex ratio measures relate to subjective experiences of partner market imbalances. Specifically, we tested the association of local sex ratios with singles’ reported surplus encounters with individuals of their own sex. Combining survey data with German administrative population data enabled us to create a range of local sex ratio measures based on different definitions of relevant age groups and different levels of aggregation (states, counties, and municipalities) to replicate the variation in operationalizations of sex ratios in the literature. Adjusting for a number of relevant control variables, we addressed widespread, yet untested, assumptions regarding the correspondence of subjective partner market experiences and local sex ratios. To substantiate the results, a longitudinal event history analysis demonstrated the predictive validity of the subjective indicator for transitions into relationships. Together with this complementary analysis, we explored how closely local sex ratio measures capture conscious experiences of partner markets.

## Measuring the Sex Ratio

A key assumption in the literature on sex ratios is that individuals modify their behavior in response to imbalanced sex ratios. Therefore, any sex ratio measure should approximate the subjective partner market experience to be a meaningful representation of individuals’ conscious mating behavior constraints. However, the literature has been inconsistent with regard to which population subgroups should be included when calculating local sex ratios.

In particular, the age limits used to calculate sex ratios differ between studies. Use of the adult sex ratio (ASR) for the 16- to 40-, 45-, or 50-year-old population is very common in the literature (Schacht & Kramer, 2016; Schacht & Smith, 2017; Schacht et al., 2016; Uggla & Mace, 2017). Some studies have extended this range to include adults up to age 64 (Barber, 2000; Kruger et al., 2010; Lippa, 2009). A variant of ASR is the operational sex ratio (OSR), which only includes unmarried or single adults (Kruger et al., 2010; Kruger & Schlemmer, 2009a, b). However, excluding partnered individuals from sex ratios to capture partner markets overlooks the fact that, especially in Western societies, divorces are common and infidelity remains among the leading causes for divorce (Amato & Previti, 2003; Rapp et al., 2015; Scott et al., 2013; South & Lloyd, 1995). Therefore, married and partnered individuals are not necessarily irrelevant to the partner market, as OSRs would imply. Accordingly, OSRs appear theoretically inferior to ASRs when approximating subjective partner markets. Moreover, in monogamous societies, differences between OSRs and ASRs solely reflect long-distance relationships and noncohabiting marriages. Consequently, scholars have reported that in monogamous societies, ASRs and OSRs are highly correlated (Fossett & Kiecolt, 1991; Pollet & Nettle, 2008).

The majority of sex ratio studies based their analyses on only a single ASR measure (see Schacht et al., 2014:218; Pollet et al., 2017:2 for overviews). Consequently, studies commonly have assigned the same ASR to all individuals within the same regional entity. Scholars explain their choice of relevant age ranges by referring to (early) adulthood as the prime life stage for mate choice and reproduction (Kruger et al., 2010; Pollet & Nettle, 2008; Schacht & Kramer, 2016; Uggla & Mace, 2017). The intention is that ASRs reflect partner market constraints during the life stages of union formation and fertility. However, computing sex ratios for wide age brackets may blur partner market squeezes within certain age cohorts. A potentially skewed sex ratio in one age cohort might be counterbalanced by an opposite skew in other cohorts that are otherwise only of limited relevance as potential partners. This becomes problematic when using wide age brackets. 20-year-olds might occasionally enter into a relationship with a 30-year-old individual. Yet, 50- or 60-year-old partners have been shown to be the exception rather than the rule for people in their twenties (Esteve et al., 2009; Feighan, 2018; Kolk, 2015; Van Poppel et al., 2001). Hence, sex ratios for wide age brackets should only be loose approximations of subjective partner markets since including less-relevant age cohorts introduces random noise in the measure.

Some authors have approached this problem by using narrower age brackets and by incorporating age heterogamy patterns into sex ratio measures by using age shifts between female and male cohorts (Parrado & Zenteno, 2002). Age hypergamy, in which men are approximately two years older than their female partners, is a demographic constant in many populations (Esteve et al., 2009; Feighan, 2018; Kolk, 2015; Van Poppel et al., 2001). Sex ratios using age shifts reflect this age discrepancy by including male age cohorts that are older than their female counterparts. A more sophisticated approach that has been proposed in the literature is to weight sex ratios based on the existing age and educational homogamy patterns to obtain “availability ratios” (Eckhard & Stauder, 2017; Fossett & Kiecolt, 1991; Goldman et al., 1984). However, availability ratios have been criticized for having problems of endogeneity since hyper- or hypogamy may themselves be responses to unbalanced partner pools (De Hauw et al., 2014).

The current study analyzed the correlation between multiple operationalizations of local sex ratios and subjective surpluses of encounters with same-sex individuals from a nationwide survey. To replicate the variety of operationalizations of local sex ratios in the literature, a range of local sex ratio measures were calculated from German administrative population data. These measures reflected different definitions of relevant age groups for different levels of aggregation (states, counties, and municipalities). Past studies have shown that future partners tend to live in proximity to one another; 85% of couples in Germany live within 20 km of one another before forming a relationship (Lengerer, 2001:142). Although the recent rise of Internet dating might have obliterated regional partner market boundaries, recent studies have shown that online dating markets and social networks exhibit a considerable degree of regional clustering (Bailey et al., 2018; Bruch & Newman, 2019). Consequently, the regional nature of partner markets should still have been meaningful during the study period (2008–2015). In Germany, counties are responsible for organizing public transport and schools, which results in a noticeable clustering of social life into these entities. Therefore, counties should approximate local partner markets better than smaller (municipalities) or larger (states) entities (Eckhard & Stauder, 2017; Obersneider et al., 2018). Consequently, county-level sex ratios should exhibit the closest relation to reports of surplus encounters with same-sex individuals, compared with sex ratios for other regional entities. Moreover, age-specific sex ratios should better capture partner market imbalances and therefore correlate more closely with subjective reports of surplus encounters with same-sex individuals than adult sex ratios since they approximate age bounds of partner pools more adequately. Generally, if local sex ratios were closely associated with subjective partner market experiences, women should be less likely to report meeting predominantly other women as the local sex ratios becomes more male-skewed. For men, on the other hand, we expected a positive association between male-skewed sex ratios and reported surplus encounters with other men.

## Methods and Data

This analysis combined data from two sources: First, individual data on subjective partner market experience and individual-level control variables came from the first seven waves (2008–2015) of the Panel Analysis of Intimate Relationships and Family Dynamics (pairfam) (Brüderl et al., 2018). Pairfam is an annual panel survey of partnership and family dynamics in three German birth cohorts (1971–1973, 1981–1983, and 1991–1993). In total, 12,402 respondents participated in the first wave of the survey. These data are particularly suited for this study given the stratified two-stage sampling procedure (Brüderl et al., 2016). In the first stage, municipalities of the Federal Republic of Germany were sampled. Subsequently, respondents were randomly selected based on local population registers, resulting in a stratified random sample that matched the hierarchical nature of this analysis (individuals being clustered into regional entities). Second, local sex ratios were calculated from official population data for German states (NUTS level 1), counties (NUTS 3), and municipalities (LAU 1) as published by the Federal and Regional Statistical Office (table series 173–43 and 173–44).

### Surplus of Encounters with Same-Sex Individuals

The focal indicator of this study, the surplus of encounters with same-sex individuals, captures whether respondents think they meet (far) more individuals of their own sex than of the opposite sex (see Table 1 for the exact phrasing). On the original scale, scores of 4 or 5 were indicative of a high surplus of interactions with same-sex individuals relative to opposite-sex individuals. We dichotomized the original scale to reflect whether respondents report such a marked subjective surplus of same-sex encounters (0) or not (1). We chose this approach over a linear model to remove potential noise in categories 1 to 3. Specifically, respondents who met an equal number of men and women might have responded “Not at all” (1) to document their disagreement with the question’s statement or picked category 3 to convey their experience of a balanced sex ratio. Linear regression models using the full five-point scale were fitted as robustness checks. Similarly, we ran multinomial regression models predicting agreement (answer categories 1 and 2) and disagreement (4 and 5) with the indicator statement, relative to the middle category (3). These models helped to evaluate the hypothesized cutoff point between categories 3 and 4. Results from these models are available in the Electronic Supplementary Material (ESM).

**Table 1:** Distribution of the indicator on surplus of encounters with same-sex individuals

Dichotomous indicator	0			1	
Original answer	Not at all	2	3	4	Absolutely
Male respondents: “I meet far more men than women”	17.9	23.5	28.3	21.2	9.1
Female respondents: “I meet far more women than men”	20.1	25.5	27.1	17.6	9.7

Values are percentages

The indicator question was part of the interview section for respondents who identified as singles in a preceding question. Interviewers prefaced the section with the following phrase: “And now to your chances of meeting a partner. To what extent do the following statements apply to your situation?” Consequently, interviewers directed respondents toward reflecting on interactions relevant to their subjective experience of the partner market when answering the indicator question. Table 1 displays the distribution of the subjective indicator and the original wording of the indicator question.

Since the indicator on opposite-sex encounters was part of pairfam’s survey module for singles, the analytical sample was restricted to respondents who were not in a relationship at the time of the interview. Furthermore, because of filtering questions, the sample has been also restricted to those singles who do not report that they are uninterested in having a partner. Moreover, the analytical sample was restricted to heterosexual individuals because the sex ratio literature focuses on heterosexual individuals, and homosexual relationships only constitute .3% of couples in Germany (Eckhard & Stauder, 2018).

### Local Proportions of Men

Local sex ratios were calculated from official annual population data for German states (Länder), counties (Landkreise and Kreisfreie Städte), and municipalities (Gemeinden) as published by the Federal and Regional Statistical Office (table series 173–43 and 173–44). Individuals from the pairfam survey were linked with local sex ratios based on their municipality of (primary) residence. This created a unique combination of the nationwide variation in local sex ratios with detailed individual-level survey data. Official population data was per December 31, and the pairfam survey interviews were conducted in the autumn and winter of the respective year, which allowed a close chronological match of both measurements. Following the recommendations of Ancona et al. (2017), this analysis operationalized sex ratios as the proportion of the male population (PM) for the respective age groups and administrative entities.

Instead of sex ratios being calculated for the single population, the local sex ratio measures included the entire population of the respective entity for three main reasons. First, married and partnered individuals may leave their current partner for a new relationship and therefore are not permanently removed from the partner market. In fact, research has shown that the availability of alternative partners increased the likelihood of union dissolutions, and infidelity remains among the leading causes for divorce (Amato & Previti, 2003; Rapp et al., 2015; Scott et al., 2013; South & Lloyd, 1995). Moreover, correlations between the sex ratio for unmarried individuals and the sex ratio for the entire population of the same age bracket are very high (e.g., Fossett & Kiecolt, 1991). This also makes sense from a theoretical point of view: sex ratios can only differ when married individuals are not living in the same regional entity as their spouses, a relationship constellation that is rare in Germany (Dorbritz & Naderi, 2013). Lastly and most importantly, the subjective indicator question did not differentiate between encounters with married and single individuals. Consequently, including all persons when calculating the local sex ratios appeared appropriate to ensure an optimal match of the subjective indicator on surplus interactions with same-sex individuals and the local sex ratio measures for the analysis.

This study used two types of local sex ratio measures: first, the proportion of men in the adult population aged 16 to 39, 16 to 49, and 16 to 64 for the respective geographies replicated adult sex ratios as commonly used in previous research (henceforth referred to as PMA). The age range of 16 to 64 is most likely too large to be a meaningful measure for partner markets. It therefore mainly served as a reference point, despite its occasional use in the literature (e.g., Barber, 2000; Kruger et al., 2010; Lippa, 2009). A second variant was the age-specific proportion of men in the population (ASPM) which reflected the proportion of men in a certain age group, including those that are one, two, three, four, or five years younger or older than the individual. ASPM can be understood as an adaptation of availability ratios, but it avoids endogeneity problems because of the weighting of sex ratios based on existing heterogamy patterns (De Hauw et al., 2014:11). For example, for a person aged 30, an ASPM with a 4-year age-width reflects the sex ratio of the 26- to 34-year-old population. Moreover, the analysis also tested ASPMs with age shifts that reflect men who are one and two years older than their female counterpart groups to incorporate the typical age hypergamy in Germany (Klein, 1996). Here, an ASPM with a 4-year age-width and a 2-year age shift would have resulted in a 30-year-old man being assigned the sex ratio of 26- to 34-year-old men paired with 24- to 32-year-old women. The 4-year-wide ASPM for women aged 30 incorporated 28- to 36-year-old men and 26- to 34-year-old women. Due to data limitations, ASPMs were only obtainable for counties and states. Overall, the variance of sex ratios is smaller when the geographical units are larger and when more age cohorts are included in the sex ratio measures (Tables 2 and 3). The PMAs vary less than the ASPMs, and sex ratios for states vary less than those for counties, which in turn exhibit less variation than municipality-level sex ratios (Tables 2 and 3).

**Table 2:** Proportion of men in the adult population (PMA) by administrative level for individuals in the pairfam sample, 2008–2014

Admin. level	Age cohorts	Mean	SD	Min	Max
State	16–39	51.05	1.07	49.16	53.43
	16–49	50.99	0.80	49.81	52.75
	16–64	50.58	0.56	49.62	52.00
County	16–39	51.11	1.47	46.32	55.47
	16–49	51.02	1.09	47.44	54.22
	16–64	50.60	0.87	47.85	53.22
Municipality	16–39	51.13	1.84	38.79	60.26
	16–49	51.06	1.49	45.18	58.67
	16–64	50.57	1.21	44.90	55.62

Values are percentages

**Table 3:** Age-specific proportions of men (ASPMs) for individuals in the pairfam sample, 2008–2014

		State-level				County-level			
Age shift	Cohort width	Mean	SD	Min	Max	Mean	SD	Min	Max
0	± 1	51.21	1.06	47.84	54.72	51.29	1.88	40.56	58.31
	± 2	51.21	1.05	47.73	54.54	51.29	1.81	41.48	57.98
	± 3	51.22	1.02	47.87	54.36	51.29	1.76	42.37	57.41
	± 4	51.22	1.00	48.04	54.18	51.29	1.72	43.36	57.20
	± 5	51.22	0.98	48.21	54.16	51.29	1.67	44.01	56.85
1	± 1	52.25	1.65	48.16	58.66	52.32	2.06	43.70	62.89
	± 2	52.11	1.50	48.40	56.51	52.18	1.87	44.02	59.86
	± 3	51.92	1.32	48.28	55.54	51.98	1.70	44.57	59.28
	± 4	51.75	1.18	48.28	54.62	51.79	1.58	45.09	58.50
	± 5	51.64	1.08	48.51	54.22	51.69	1.51	44.70	57.86
2	± 1	52.98	2.48	47.74	64.14	53.05	2.85	44.51	68.88
	± 2	52.77	2.20	48.25	60.96	52.82	2.53	44.40	64.88
	± 3	52.51	1.86	48.59	58.50	52.56	2.17	44.42	61.82
	± 4	52.26	1.61	48.49	56.75	52.30	1.91	44.11	60.69
	± 5	52.06	1.41	48.67	55.75	52.09	1.72	44.15	59.49

Values are percentages

### Statistical Approach

Our analytical approach consisted of two parts: First, the data for all seven waves was pooled to analyze the association between the subjective surpluses of encounters with same-sex individuals and PMAs or ASPMs. To incorporate the nested data structure, three-level multilevel regression models were fitted. The dependent variable on level one was the reported surplus of encounters with same-sex individuals. To adjust for unmeasured variation between individuals on level two and administrative units (i.e., states, counties, and municipalities) on level three, two random intercepts were included. Separate models for each measure of the local proportion of men were fitted. All PMA and ASPM measures were *z*-standardized to yield partially standardized coefficients (Menard, 2004). Consequently, all results presented below convey the difference in the probability to report a surplus of own-sex contacts associated with a one-standard-deviation increase in the respective PMA or ASPM.

In a second analysis, we explored how both local sex ratios, operationalized as PMA and ASPM, and the surplus of encounters with same-sex individuals predicted relationship formation using longitudinal multilevel discrete-time event history models. The occurrence of relationship formation is time-lagged to avoid reverse causality. We used logistic regression models including random intercepts for the administrative units to predict the probability of finding a partner in the subsequent panel wave. Again, separate models for each of the local PMA and ASPM measures were calculated and all continuous variables were *z*-standardized.

Table 4 shows that our analytical sample included 3341 men and 2686 women for the regression models at the state level. Due to missing population data resulting from administrative reorganizations during the observation period, the samples for counties and municipalities were slightly smaller than for states. The longitudinal sample comprised 2573 men and 2041 women with 4532 years at risk for women and 6655 years at risk for men. During the observation period, 1249 women and 1274 men formed a new relationship. All models adjusted for a set of individual and contextual control variables. On the individual level, these were age and age squared, (being on track for) higher secondary education (i.e., Abitur), labor force status and parental status. Contextual control variables included residence (i.e., in East or West Germany) and the population size of the residential municipality. Models were fitted separately for men and women.

**Table 4:** Sample sizes for analytical models

		Regression Sample		Event History Sample		
	Unit	Singles	Person-years	Singles	Person-years	Relationships formed
Women	State	2606	6029	2041	4532	1249
	County	2604	6022	2039	4525	1246
	Municipality	2603	6025	2040	4530	1249
Men	State	3268	8817	2573	6655	1274
	County	3265	8807	2571	6647	1272
	Municipality	3267	8814	2572	6653	1273

Sample sizes for county-level sex ratio models were smaller than for municipality-level models due to missing population data resulting from an administrative restructuring in the state of Mecklenburg-Vorpommern in 2011

In addition to conventional significance testing of coefficients, the effectiveness of the respective local sex ratio measure for predicting subjective surpluses of encounters with same-sex individuals was assessed based on the area under the receiver operating characteristic (ROC) curve (AUC). The AUC ranges from .5 to 1 and summarizes a model’s ability to discriminate between those subjects who experience the outcome of interest versus those who do not (Hosmer et al., 2013:177). Chi-square tests of areas under the curve helped to assess whether adding a PMA or ASPM improved the discrimination relative to a null model with the same specifications and control variables but without any local sex ratio measure (Cleves, 2002). Intraclass correlations (ICCs) were calculated for each level of measurement to reveal the potential clustering of individuals in the dataset across states, counties, and municipalities. The ICC describes how strongly those within a group resemble one another based on the proportion of the variance accounted for by the group level (Snijders & Bosker, 2012:17).

Due to the large number of separate models, results are presented as combined coefficient plots that only display coefficients for the focal sex ratio variable from each model. Given the strong directional expectations, the coefficient plots display both conventional 95% as well as 90% confidence intervals to indicate results for one-tailed comparisons. Detailed results tables, including coefficients for the control variables, can be found in the supplementary material (ESM).

## Results

### Surplus of Same-Sex Encounters and Local Male Population Shares

**Fig. 1:**
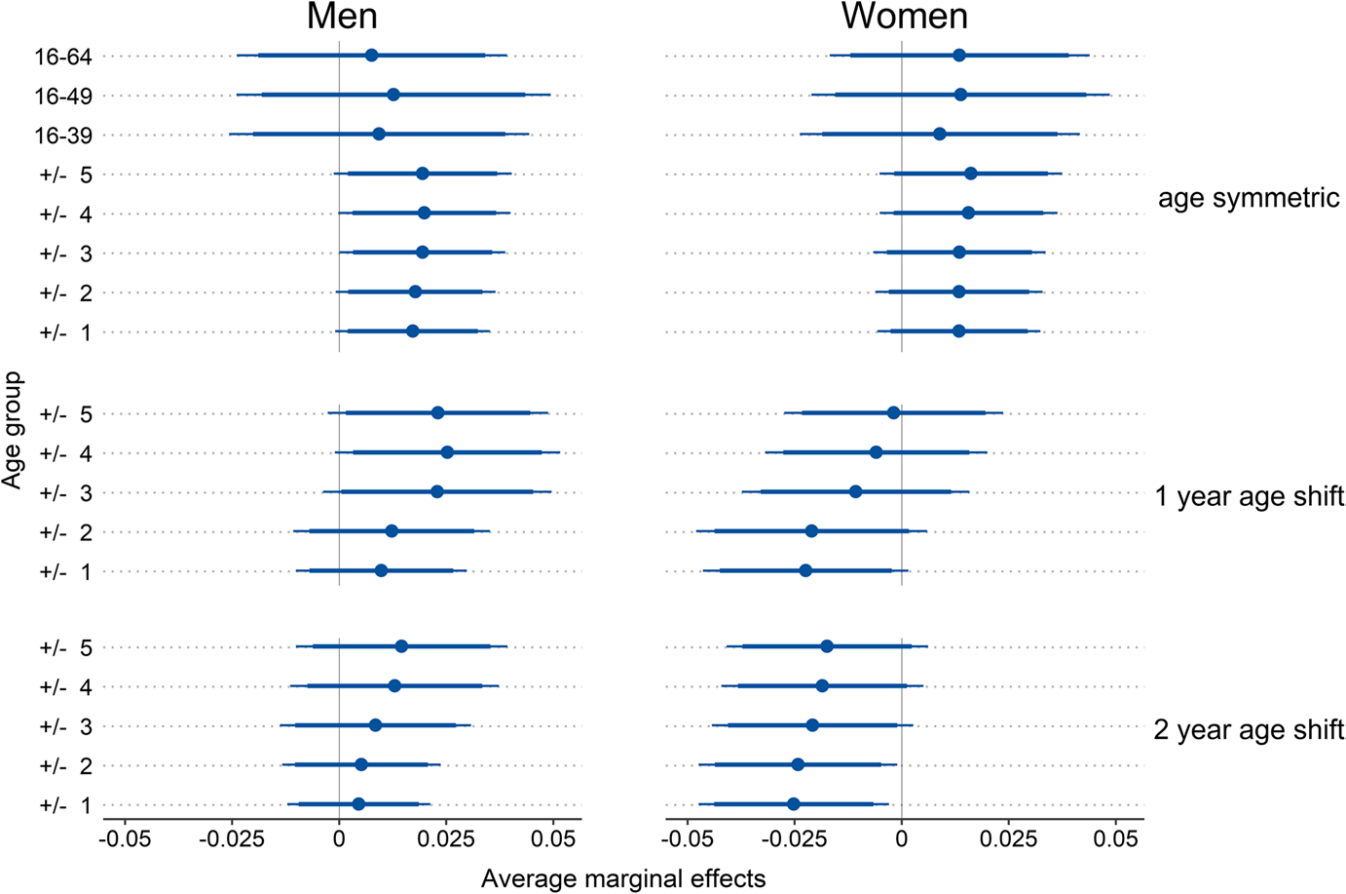
Average marginal effects for the prediction of reported surpluses of encounters with same-sex individuals from state-level proportions of men. Models included random intercepts for individuals & states and adjusted for age, age^2^, education, East/West Germany, size of residential municipality, employment status, and parental status. Error ranges indicate 90% confidence intervals (CIs; thick horizontal lines) and 95% CIs (thin horizontal lines). *N* (men) = 8817; *N* (women) = 6029. ICC (men) = 0.0046; ICC (women) = 0.0089

Figure 1 displays the partially standardized average marginal effects of state-level proportions of men on the probability to report a surplus of encounters with same-sex individuals (SESSI) from a series of logistic regression models. The panels on the right reveal that at the state level, only selected age-specific proportions of men that were age-shifted were significantly associated with SESSIs as reported by female respondents. In particular, none of the indicators for proportions of men in the adult population (PMA) yielded significant associations with SESSIs as reported by female respondents. Similarly, age-specific proportions of men (ASPMs) without age shifts were not significantly associated with female respondents’ SESSI either (upper right panel of Fig. 1). This finding was consistent for all ASPMs representing populations that are one, two, three, four, or five years younger or older than the individual. Estimates for both age-symmetric ASPMs and PMAs were not only nonsignificant but also contrary to theoretical expectations. Coefficients suggested that women were more likely to report surplus encounters with other women as the ASPM or PMA became more male-skewed. Age-shifted ASPMs, on the other hand, suggested the opposite and more intuitive conclusion of a negative association. Nevertheless, the coefficients were again mostly nonsignificant (center and lower right panel of Fig. 1). Age-shifted ASPMs were operationalized so that female age cohorts were one or two years younger than their male counterparts to approximate common age hypergamy patterns. However, none of the ASPMs shifted by one year yielded significant associations at the conventional 5% level, and only the measure based on a one-year age bandwidth was significantly associated with women’s SESSI at the 10% level (center right panel of Fig. 1). ASPMs with a one- or two-year age bandwidth that were age-shifted by two years yielded significant associations (lower right panel of Fig. 1): women were becoming less likely to report that they primarily meet other women since men aged two to four (one to five) years older increasingly outnumbered women that were one year (two years) older or younger than the focal female respondent. Among the other ASPMs with a two-year age shift, the ±3 ASPM yielded a coefficient that is significant at the 10% level, whereas broader age ranges did not yield significant correlations with women’s reports of primarily encountering women.

For men, none of the state-level PMAs and only selected ASPMs were significantly associated with men’s reports of surplus encounters with other men (left-hand panels of Fig. 1). All coefficients for PMAs and ASPMs were positive in sign, which is in line with theoretical expectations: men were more likely to report that they predominantly met other men when the state’s population was more male-skewed. However, PMAs in particular yielded estimates that were nonsignificant (upper left panel of Fig. 1). Moreover, coefficients for age-symmetric ASPMs were essentially the same size. Yet, all but the ASPM including a state’s population aged three years older or younger were too wide to reach statistical significance at the 5% level and thus were only significant at the 10% level. ASPMs with a one-year age shift with a bandwidth larger than 3 years yielded coefficients that were only significant at the 10% level, while narrower ASPMs also failed this level of significance (center left panel of Fig. 1). We did not find any significant associations at the 5% level of significance for ASPMs incorporating a two-year age shift (bottom left panel of Fig. 1). Notably, the intra-class correlations suggested that reports of surplus encounters with same-sex individuals did not correlate within states among either women or men.

**Fig. 2:**
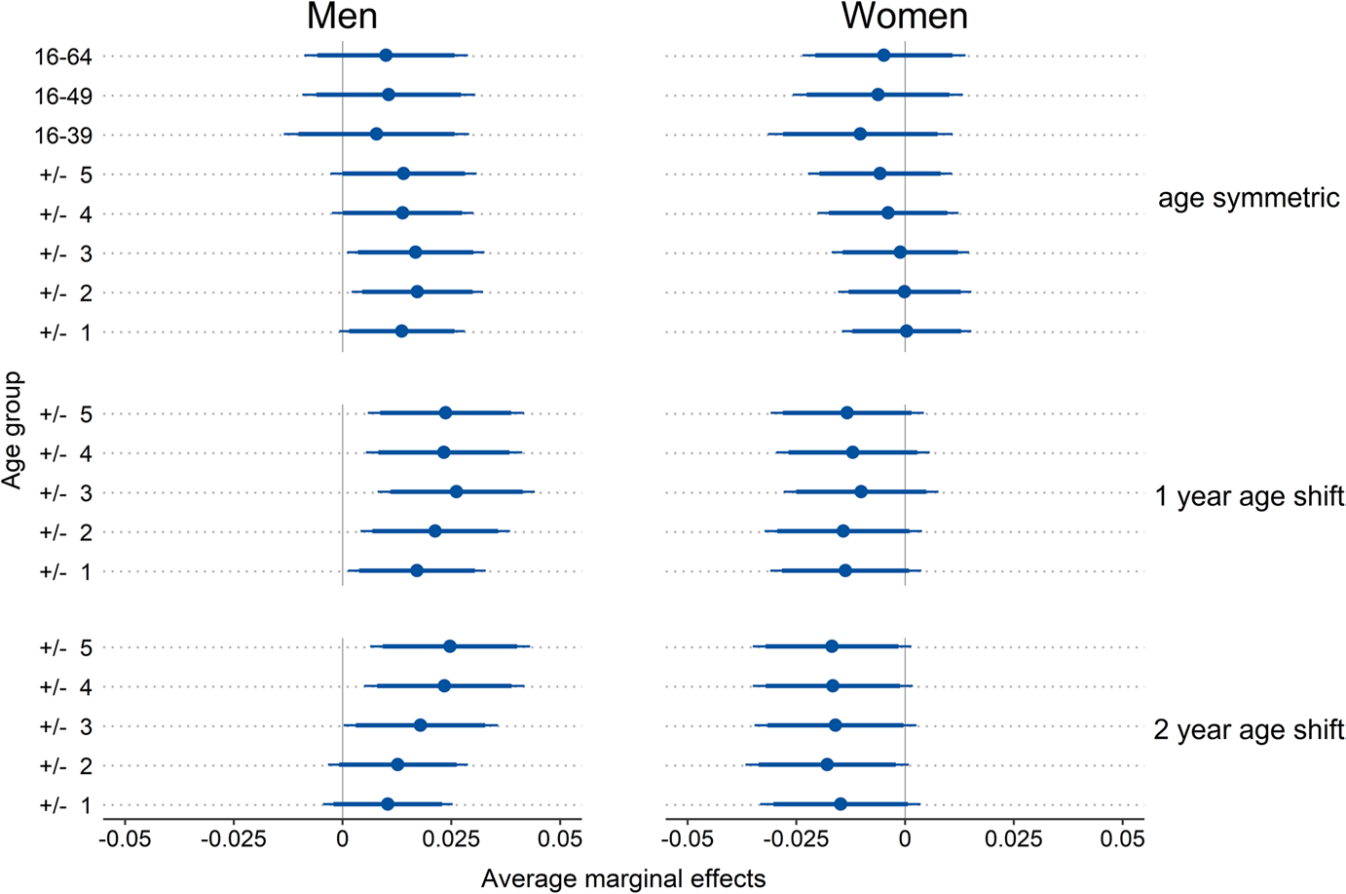
Average marginal effects for the prediction of reported surpluses of encounters with same-sex individuals from county-level proportions of men. Models included random intercepts for individuals & counties and adjusted for age, age^2^, education, East/West Germany, size of residential municipality, employment status, and parental status. Error ranges indicate 90% confidence intervals (CIs; thick horizontal lines) and 95% CIs (thin horizontal lines). *N* (men) = 8807; *N* (women) = 6022. ICC (men) = 0.0311; ICC (women) = 0.0254

Results for smaller entities supported the notion that age-specific measures of the proportion of men (ASPM) proved to be better predictors than measures that include all adult age cohorts (PMA) when predicting surplus encounters with same-sex individuals (SESSI). For both men and women, county-level PMAs failed to predict SESSI in a statistically significant way (upper three coefficients in top panel of Fig. 2). On the other hand, ASPMs yielded significant associations with men’s SESSI. Age-symmetric ASPMs including the population that is two or three years younger or older than the individual yielded coefficients correlated at the 5% level with men’s SESSI. Furthermore, age-symmetric ASPMs both wider and narrower reached the 10% level of significance (upper left panel of Fig. 2). Moreover, all ASPMs that were age-shifted by one year were significantly associated with men’s SESSI at the 5% level (center left panel of Fig. 2). Similarly, men’s SESSI was significantly associated with ASPMs that were age-shifted by two years and included the population that is three, four, or five years older or younger than the individual. For women, however, ASPMs with a two-year age-shift only yielded coefficients that were significant at the 10% level. ASPMs that were age-symmetric or shifted by one year did not predict women’s SESSI in a statistically significant way (right-hand panels of Fig. 2). The ICCs suggested that the correspondence of SESSI within the same county was slightly higher than within states, but again it was negligible.

**Fig. 3:**
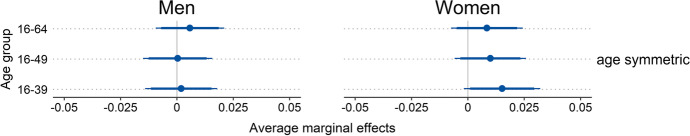
Average marginal effects for the prediction of reported surpluses of encounters with same-sex individuals from municipality-level proportions of men. Models included random intercepts for individuals & municipalities and adjusted for age, age^2^, education, East/West Germany, size of residential municipality, employment status, and parental status. Error ranges indicate 90% confidence intervals (CIs; thick horizontal lines) and 95% CIs (thin horizontal lines). *N* (men) = 8814; *N* (women) = 6025. ICC (men) = 0.0265; ICC (women) = 0.0260

Finally, none of the municipality-level PMAs yielded coefficients that were significant at the 5% level (Fig. 3). Note that estimates for women were also counterintuitive in sign. Estimates suggested a positive association between surplus encounters with other women and a surplus of men in the residential municipality. However, although the PMA including adults aged 16 to 39 reached only the 10% level of significance, none of the coefficients suggested any statistically significant association. Again, the intra-class correlations of individuals’ SESSI from the same municipality were very low for both men and women.

Overall, ASPMs yielded marginal effect estimates that were mostly larger than those for the PMAs and also corresponded to theoretical expectations. In particular, age-shifted ASPMs resulted in larger effect estimates, with selected measures also reaching statistical significance at the 5% level. However, both PMAs and ASPMs only marginally improved the predictions of SESSI as measured by the area under the ROC curve (AUC) (Fig. S1, in the ESM). Across all levels of aggregation, the AUC does not exceeded .6, which is generally considered a low model fit (Hosmer et al., 2013). In particular, none of the state-, county-, or municipality-level PMAs improved predictions for men’s or women’s SESSI. State-level ASPMs did not significantly improve model fit relative to the respective null model that included all of the control variables but none of the sex ratio measures. This was consistent for both men and women and even included those state-level ASPMs that yielded significant coefficients. County-level ASPMs improved predictions only when age-shifted and only for men’s, but not women’s, SESSI. By a small margin, the ASPM that included the county’s population three years older or younger than the respondent and incorporated a one-year age shift yielded the best model fit for men.

Finally, the general finding of weak associations of ASPMs with SESSI and even weaker ones for PMAs was also supported by auxiliary results. Fig. S2 (in the ESM) displays the correlation coefficients for the pooled bivariate association between local sex ratio measures and the subjective indicator. None of the correlations exceeded an absolute value of .053. Bivariate correlations did not adjust for correlations of SESSIs within individuals and administrative entities. This likely resulted in an overestimation of correlation coefficients. Consequently, the already low correlation coefficients constitute an upper bound of the true association. Moreover, linear models based on the original 5-point Likert scale support the overall impression of a weak association of local PMAs and ASPMs with SESSI. Compared with the logistic regression results, the linear models yielded fewer significant coefficients (Figs. S3–S5 in the ESM). As discussed above, the differences between both models might have been due to noise in response categories 1–3. Results from multinomial regression models using a three-category variant of the original indicator scale further corroborated this impression (Figs. S6–S8 in the ESM). Specifically, probabilities of (strong) agreements with the subjective indicator statement were more sensitive to PMAs and ASPMs than the probabilities of disagreement. Relative to the undecided answer category (3), we found significant marginal effects for the probability to agree with the original indicator statement (4 or 5) that closely resemble those for the logistic models presented above. Yet, none of the state- and county-level PMAs or ASPMs yielded significant marginal effects for the probability to disagree with the indicator statement (1 or 2). We did find one significant marginal effect of PMAs on the probability to disagree with the indicator statement. Disagreement with the indicator statement suggests that women meet more men than women. Thus, women should be more likely to disagree with the statement when they are living in male-skewed municipalities. However, the coefficient was contrary to these theoretical expectations: women were less likely to report meeting more men than women as the municipality-level population became more male-skewed. Yet overall, multinomial regression results suggested no systematic differences between undecided and disagreeing answers. Thus, multinomial regression results supported the approach to dichotomize the original scale using a cutoff point of 3 for more parsimonious logistic models.

Table S1 (in the ESM) shows the logistic coefficients of the control variables, which remained essentially the same across all models. Similarly, analyzing the data for each wave separately yielded essentially the same results as those presented here.

### Relationship Formation

**Fig. 4:**
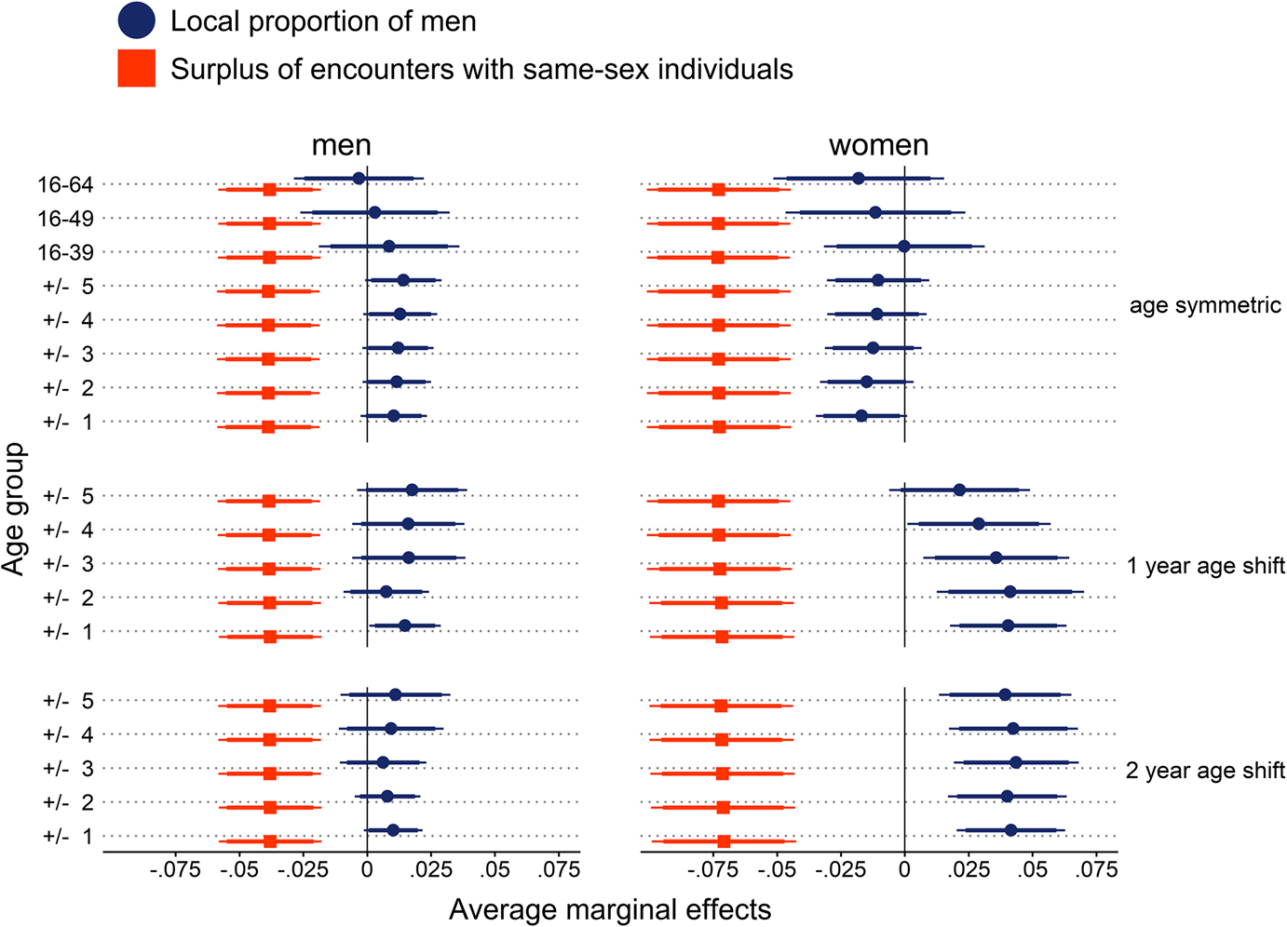
Average marginal effects of surpluses of encounters with same-sex individuals (SESSI) and state-level proportions of men on relationship formation from logistic discrete-time event history models predicting relationship formation. Coefficients for local proportions of men reflect changes in the probability to enter a relationship for a one standard deviation increase in the male population share. Coefficients for surplus encounters with same-sex individuals reflect changes in the probability to enter a relationship when reporting a surplus of encounters with individuals of one’s own sex compared to undecided or negative answers. Models included random intercepts for individuals & states and adjusted for age, age^2^, education, East/West Germany, size of residential municipality, employment status, and parental status. Error ranges indicate 90% confidence intervals (CIs; thick horizontal lines) and 95% CIs (thin horizontal lines). *N* (men) = 6655; *N* (women) = 4532. ICC (men) = 0.0002; ICC (women) = 0.0013

Figure 4 shows the average marginal effects of the indicator on surplus of encounters with same-sex individuals (SESSI) as well as the state-level proportions of men among adults (PMA) and age-specific male population shares (ASPM). For both men and women, SESSI was significantly and negatively associated with relationship formation. In other words, the probability of finding a partner in the subsequent panel wave was lower if the respondents reported a higher surplus of interactions with individuals of their own rather than the opposite sex. The average marginal effect of SESSI for women was nearly twice as large as the effect for men, suggesting a greater relevancy of surpluses of same-sex interactions for women than for men.

With regard to the local sex ratio indicators, none of the state-level PMAs were associated with men’s probability to enter a relationship. Age-symmetric ASPMs proved to be significant predictors of male relationship formation only at the 10% level. Age-shifted ASPMs proved to be only slightly better predictors. Among ASPMs with a one-year and two-year age shift, only those that included the population one year older or younger than the individual yielded coefficients there were significant at the 5% and 10% levels, respectively. For women, the state-level ASPMs were correlated with relationship formation at the 5% level only when age hypergamy was incorporated. The probability of females finding a partner was higher when there was a higher proportion of men within a state. Similar to the findings for men, state-level PMAs were not significantly correlated with female relationship formation. Overall, the ICC values were low, suggesting no correlation of the probability to form a relationship within states.

**Fig. 5:**
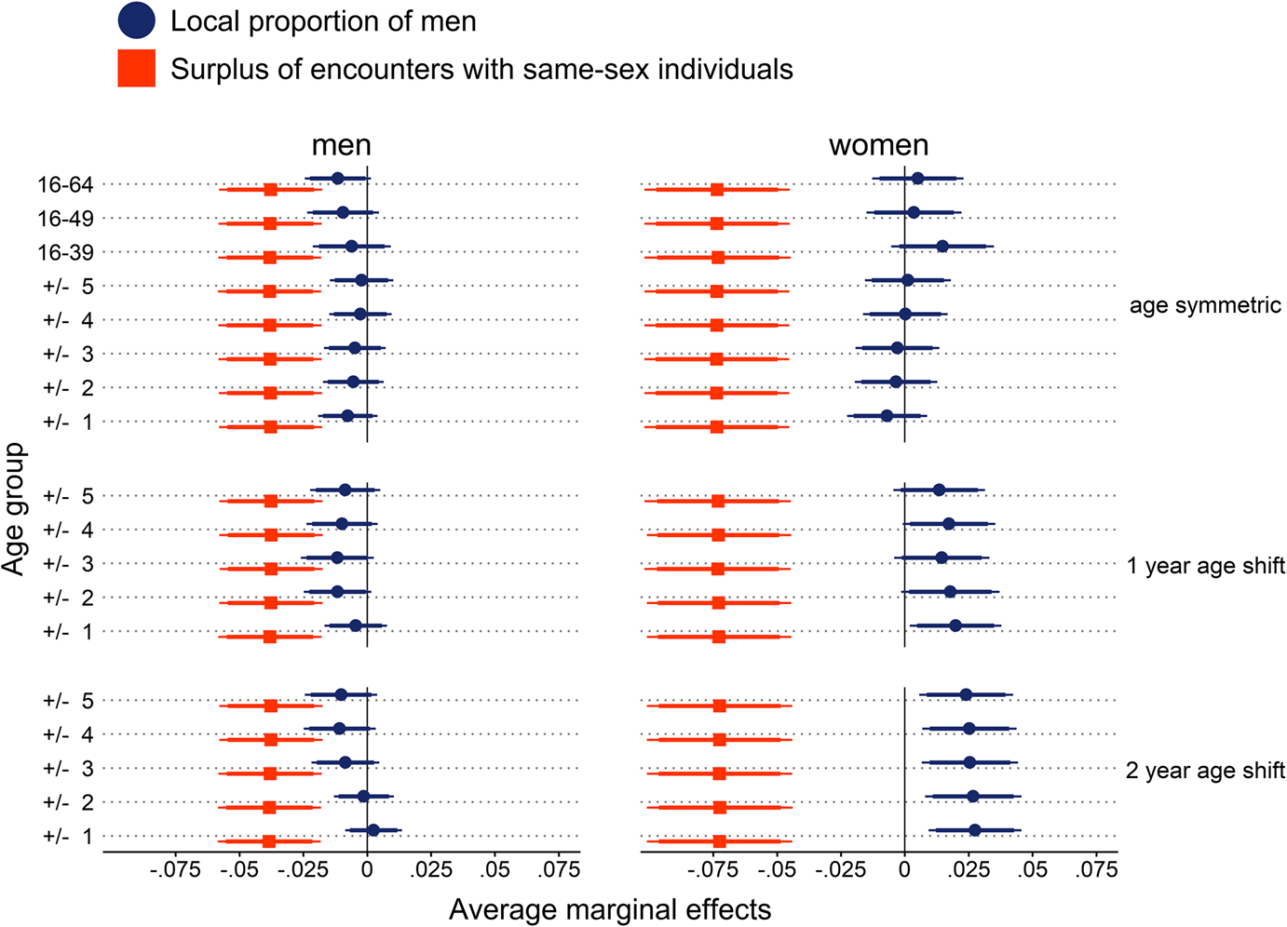
Average marginal effects of surpluses of encounters with same-sex individuals (SESSI) and county-level proportions of men on relationship formation from logistic discrete-time event history models. Coefficients for local proportions of men reflect changes in the probability to enter a relationship for a one standard deviation increase in the male population share. Coefficients for SESSI reflect changes in the probability to enter a relationship when reporting a surplus of encounters with individuals of one’s own sex compared to undecided or negative answers. Models included random intercepts for individuals & counties and adjusted for age, age^2^, education, East/West Germany, size of residential municipality, employment status, and parental status. Error ranges indicate 90% confidence intervals (CIs; thick horizontal lines) and 95% CIs (thin horizontal lines). *N* (men) = 6647; *N* (women) = 4525. ICC (men) = 0.0065; ICC (women) = 0.0161

Models using county-level sex ratio measures replicated the insignificant results for age-symmetric proportions of men (Fig. 5). For both men’s and women’s relationship formation, marginal effects of PMAs were very close to zero and failed to meet any level of significance. ASPMs yielded marginal effects on male chances to enter a relationship that were only significant at the 10% level, including those age-specific indicators incorporating an age shift. Similarly, female relationship formation was only associated with age-symmetric ASPMs at the 10% level of significance. However, all of the ASPMs with a two-year age shift—that is, female age cohorts two years younger than the corresponding male age cohorts—turned out to be significant predictors of women’s transitioning into a relationship. Specifically, results suggest that a higher age-specific and two-year age-shifted male population share within a county is associated with a higher probability for women to enter a relationship. Surpluses of encounters with same-sex individuals were consistently negatively associated with relationship formation, and differences in the size of the estimates between the models are negligible (see Table S2 for coefficients of control variables).

**Fig. 6:**
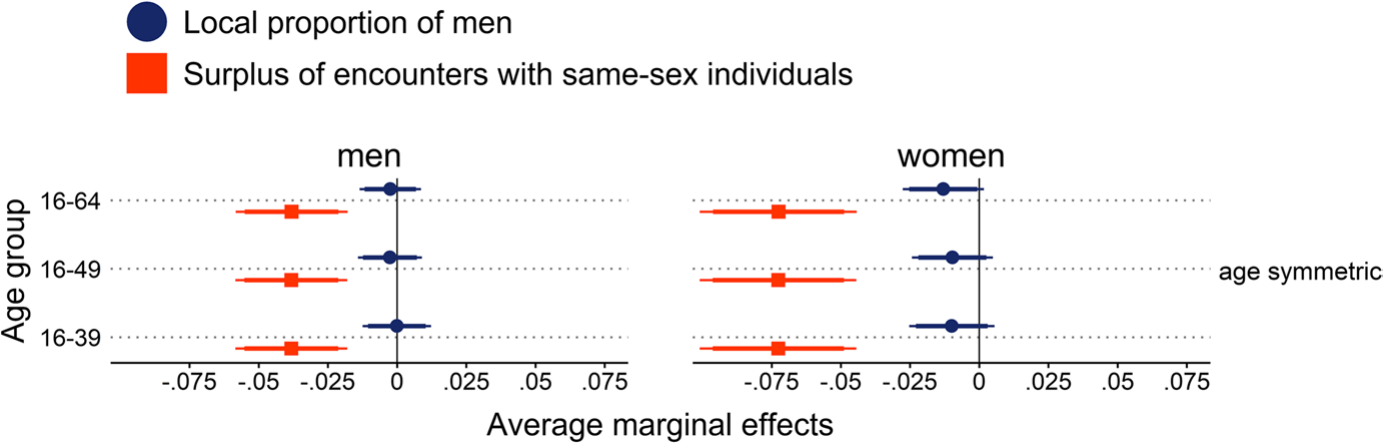
Average marginal effects for surpluses of encounters with same-sex individuals (SESSI) and municipality-level proportions of men on relationship formation from logistic discrete-time event history models. Coefficients for local proportions of men reflect changes in the probability to enter a relationship for a one standard deviation increase in the male population share. Coefficients for surplus encounters with same-sex individuals reflect changes in the probability to enter a relationship when reporting a surplus of encounters with individuals of one’s own sex compared to undecided or negative answers. Models included random intercepts for individuals & municipalities and adjusted for age, age^2^, education, East/West Germany, size of residential municipality, employment status, and parental status. Error ranges indicate 90% confidence intervals (CIs; thick horizontal lines) and 95% CIs (thin horizontal lines). *N* (men) = 6653; *N* (women) = 4530. ICC (men) = 0.0063; ICC (women) = 0.0079

Similarly, Fig. 6 shows that the coefficients for surpluses of same-sex interactions remain the same in the models that include municipality-level proportions of men among adults (PMA). As in the models for state- and county-level sex ratios, the municipality-level PMAs did not correlate significantly with the probability of female or male relationship formation. Hence, PMAs were not a significant predictor of relationship formation at any level of aggregation. Furthermore, the ICC values revealed that the correlation of transitions into relationships were actually higher when clustered by counties than by municipality. However, these differences should be treated with caution given that, overall, the ICCs were very low for any configuration.

Figure S9 (in the ESM) reveals that models that included only either the subjective indicator or the measure for local proportions of men yielded essentially the same coefficients. This underscores the lack of correlation between the SESSI indicator and local ASPMS and PMAs as presented in the previous section.

## Discussion

Imbalanced sex ratios have been linked to a wide range of social consequences, including family formation, economic decision-making, gender roles, partnership formation, fertility, personality, and sexuality (Bauer & Kneip, 2013; Feingold, 2011; Griskevicius et al., 2012; Harknett, 2008; Merli & Hertog, 2010; Pollet & Nettle, 2008; Schacht & Smith, 2017; Trent & South, 2012; Uggla & Mace, 2017). Sociodemographic and evolutionary mating market approaches have explained these findings by shifts in bargaining power based on differential mating opportunities for men and women in an imbalanced sex ratio environment (Filser & Schnettler, 2019; Guttentag & Secord, 1983; Kokko & Jennions, 2008; Pedersen, 1991; Schacht & Kramer, 2016). Individual partner market experiences might play a crucial role in these behavioral adaptations to local sex ratios given that subjective experiences provide critical guidelines for human behavior (Gilbert et al., 2016; Gintis, 2006; Kroneberg & Kalter, 2012). Yet, empirical evidence has been lacking on how closely individual experiences of partner market opportunities correspond to sex ratios of their local environment. To fill this gap, we analyzed associations between a variety of local sex ratio measures and subjective partner market experiences of female and male singles in a German panel survey.

In sum, the expected association between subjective partner market experiences and local sex ratios only held for selected, age-specific sex ratio measures. In particular, adult sex ratios based on broad age ranges as are commonly used in the literature did not prove to be significant predictors of subjective partner market experiences. This result was consistent across operationalizations of adult sex ratios as the proportion of men in the adult population (PMA) based on different age brackets at the level of states, counties, and municipalities. None of the adult sex ratio variants correlated with either men’s or women’s subjective experiences of surplus encounters with individuals of their own sex in a meaningful way. More granular, age-specific sex ratio measures (ASPM) that include only individuals of adjacent age cohorts were closer approximations of subjective partner market experiences. In particular, age-specific measures that also incorporated age shifts to reflect age hypergamy patterns proved to be better predictors of subjective partner market experiences. Nevertheless, only selected state-level, age-shifted sex ratios correlated with women’s surplus encounters with other women in a statistically significant way. The corresponding county-level age-shifted sex ratios yielded similar, yet smaller coefficients, which have to be interpreted with caution given that they did not reach statistical significance. For men, only county-level, age-shifted sex ratios significantly predicted associations with men’s subjective partner market experiences. Coefficients for state-level age-shifted sex ratios were similar in size but did not reach statistical significance. Overall, some reservations regarding the state-level findings seem warranted because the German states might be too large in geographic terms (with all but four being larger than 15,000 km^2^) to be considered a single partner market. Lengerer (2001:142) reports that 85% of future partners in Germany live within a 20 km radius of each other. Recent publications suggest that earlier recommendations to rely on smaller entities when operationalizing local partner markets continue to apply in the age of Internet dating (Bruch & Newman, 2019; Fossett & Kiecolt, 1991). Therefore, results for state-level sex ratios should be treated with caution.

In sum, the results of this study suggest that previous findings regarding the social consequences of imbalanced sex ratios are unlikely to be mediated by conscious adaptations to partner scarcities or oversupplies. Adult sex ratios for fixed age brackets, such as the population aged 16–49 or 16–64, constitute the standard operationalization of local sex ratios in the literature (see Schacht et al., 2014; Pollet et al., 2017 for reviews). Our findings suggest that sex ratios for fixed adult age ranges are unlikely to correspond closely to subjective partner market experiences. Previous research has demonstrated that sex ratios correlate only moderately with each other when different age cutoffs are used (see Fossett & Kiecolt, 1991 for a discussion using US census data). Therefore, adult sex ratios are unlikely to be a well-suited summary measure of age-specific sex ratios. This is also supported by our dissimilar results for adult and age-specific sex ratios. In contrast to adult ratios, selected age-specific and age-shifted operationalizations significantly predicted subjective partner market experiences. In particular, the integration of age hypergamy into the sex ratio measures yielded significant results for predicting subjective partner market experiences. Future research should therefore consider focusing on age-specific, age-shifted sex ratio measures. Yet, although age-shifted sex ratios predicted men’s subjective partner market experiences, we only find weak evidence for a similar association for women. This difference between men and women might be due to a smaller sample size of women in our models. A further explanation could be related to sex differences in sexual strategies guiding partner market behavior. In particular, sexual strategies theory suggests that sexual selection favored antagonistic mating competition and preferences for multiple short-term mating in men (Buss, 1999; Schmitt, 2015; Trivers, 1972). This could also entail that men are more aware of marriage squeezes than women are.

A further finding of this paper is that subjective surpluses of same-sex encounters significantly predicted relationship formation. For both sexes, a subjective surplus of encounters with individuals of one’s own sex was significantly associated with a lower probability of entering a relationship. We are aware that survey questions on subjective partner market experiences may represent an excessive demand for respondents. However, the fact that the subjective indicator correlates with this specific partner market outcome supports the idea that the analyzed reports of surplus encounters with same-sex individuals constituted a valid approximation of individual partner market experiences. Concerning the local sex ratio measures, age-specific and age-shifted variants proved to be advantageous over adult sex ratios also when predicting relationship formation. None of the adult sex ratios significantly predicted relationship formation. Moreover, age-specific local sex ratios only yielded significant coefficients when incorporating age shifts. Specifically, relationship formation for women was significantly predicted by state- and county-level age-specific and age-shifted sex ratios. Yet, the probability of men entering a relationship was not predicted by local sex ratios, replicating similar asymmetric findings by Uggla and Mace (2017). With regard to the link to subjective partner market experiences, our findings suggest that subjective partner market experiences and local sex ratios should be considered distinct context variables rather than equivalent indicators. This is even true for detailed measures of local sex ratios. For instance, age-specific county-level sex ratios with a two-year age shift were a significant predictor of women’s relationship formation. Yet, we do not find conclusive evidence that these measures were correlated with women’s subjective partner market experiences. Consequently, these findings suggest that subjective and local sex ratios are not interchangeable operationalizations. Rather, they appear to be two separate dimensions of partner market circumstances. Researchers should be aware of this distinction when offering theoretical interpretations of results based on local sex ratios.

The subjective partner market indicator used in this study is not equivalent to the situational perception of the sex proportions in a group. Instead, it approximated the everyday interactions of individuals and therefore should not be interpreted as indicative of an inability to perceive sex ratios in set groups. Both Alt et al. (2017) and Neuhoff (2017) demonstrated that participants are able to give accurate sex ratio estimations based on short-term exposure to visual and auditory cues. Against the backdrop of these previous studies, one potential explanation for our findings could be that individual partner market experiences are not a direct representation of macro-structural conditions, i.e., local sex ratios (Blau, 1977; Rapp et al., 2015; Schwartz, 1990). Instead, individual partner markets may be structured in different “foci of activity,” such as workplaces, voluntary associations, or hangouts (Feld, 1981; Rapp et al., 2015). With this in mind, studying the consequences of sex ratios in interactive spheres such as workplaces (Åberg, 2009; Barclay, 2013; Svarer, 2007), industries (Uggla & Andersson, 2018), bars (Lycett & Dunbar, 2000), or colleges (Harknett & Cranney, 2017) would have the advantage of assuming that the individuals are actually interacting with one another. This is much more plausible than the same contention would be for local sex ratios. Consequently, individuals’ foci-specific sex ratios might give a more accurate impression of partner supply and demand within the respective foci rather than sex ratios of the local population, even for their specific age cohort.

This paper used a combination of administrative population information and survey data, which is crucial to this analysis. Studies relying on such data face a trade-off between the scope of the data and the ability to link survey data with survey-based partner market measures. The pairfam survey data constitute a unique combination of both ends of this spectrum. However, adult sex ratios in Germany may not have sufficient variation to allow for identifying a clear effect. This is particularly true for adult sex ratios at the state level, which only range between 96 and 108 men per 100 women (see Table 2). Consequently, nonsignificant findings for state-level sex ratios could also be due to the lack of variation at this level of aggregation. Internationally, local adult sex ratios may vary more substantially in selected regions, most notably in the male-skewed populations of China and India (Guilmoto, 2012). However, the county-level variation in adult sex ratios in the analyzed data was consistent with that of recent studies from other Western countries (e.g., Schacht & Kramer, 2016), and the ranges of age-specific sex ratios exceeded the ranges of adult sex ratios in our data.

A further limitation is that the findings are contingent on the validity of the subjective partner market indicator. While our complementary analysis demonstrated the predictive validity of the subjective indicator with respect to relationship formation, limitations persist. The directional verbalization of the indicator question introduced ambiguity, resulting in imprecise measurement of undecided and disagreeing answers. Specifically, respondents who met an equal number of men and women either might have reported disagreeing with the statement of predominantly meeting individuals of their own sex or might have given an undecided answer to express their experience of a balanced sex ratio. We explored this issue via fitting linear and multinomial models for different variants of the original indicator scale. These auxiliary results confirmed that the difference in probabilities for undecided and disagreeing answers was not significantly correlated to local sex ratios. However, agreement with the surplus same-sex contacts scale was related to selected local sex ratio measures (Fig. S6-S8, in the ESM). We therefore focused on the dichotomized indicator that summarized disagreeing and undecided responses. Nevertheless, our logistic regression results do not persist when taking a linear modeling approach, most likely because of measurement noise in disagreeing and undecided responses. Furthermore, the current analysis was limited to one global subjective indicator of opposite-sex encounters. A detailed survey of foci-specific sex ratios might give a closer approximation of subjective partner market experiences (cf. Rapp et al., 2015). This could reveal whether partner markets in specific foci actually correspond to local sex ratios, whereas partner markets in other foci do not. In particular, detailed information on job location could be of particular relevance, given that 60% of German employees cross municipality borders when commuting (Pütz, 2017). Consequently, adding sex ratios based on the place of work could yield a higher correspondence to subjective partner markets.

In conclusion, the sex ratio literature should be cautious regarding the assumption that individuals are consciously aware of local sex ratio skews. In particular, subjective and conscious partner market experiences do not appear to be a direct function of broad-range adult sex ratios but instead are correlated only with selected, age-specific measures. Researchers should consider this when interpreting findings based on local sex ratios. Although our findings shed some doubt on a direct link between conscious experiences and local sex ratios, this does not necessarily imply that local sex ratios do not capture partner markets. So far, very little is understood about how humans experience, remember, and process contextual sex ratios (Dillon et al., 2017). In particular, the relative importance of immediate interaction partners, local communities, and broader social contexts is yet to be explored (Maner & Ackerman, 2020).

This paper explored the relationship between a general indicator of subjective partner market experiences and local sex ratio measures. In sum, general sex ratio measures that are based on broad age ranges do not seem to capture conscious partner market experiences in a meaningful way. Future research will have to establish the role of unconscious factors, including endocrinal or network effects mediating contextual local sex ratios and adaptations in individual behavior.

## Supplementary Information

ESM 1 (PDF 2.39 mb)

## Data Availability

The data used for this study are accessible to the scientific community as scientific use file from pairfam (Brüderl et al., 2018) at https://www.pairfam.de/en/data/data-access/
