# Supplementary material for: Do Local Sex Ratios Approximate Subjective Partner Markets? Evidence from the German Family Panel
Source: Hum Nat. 2021 Jun 19;32(2):406–33. doi: 10.1007/s12110-021-09397-6 (PMC8321994; doi:10.1007/s12110-021-09397-6)
Supplement: Supplementary file 1 — (PDF 2.39 mb) [file 12110_2021_9397_MOESM1_ESM.pdf]

Do local sex ratios approximate subjective partner markets? Evidence from the German Family panel

Human Nature

Andreas Filser* & Richard Preetz*

* *Institute for Social Sciences*
University of Oldenburg
Ammerlaender Heerstr. 114-118,
D-26129 Oldenburg, Germany

Author for correspondence (andreas.filser@uol.de).

Supplementary Material

https://doi.org/10.1007/s12110-021-09397-6

Fig. S 1: Model fit as measured by area under the ROC curve (AUC) for logistic regression models predicting SESSI from local proportions of men

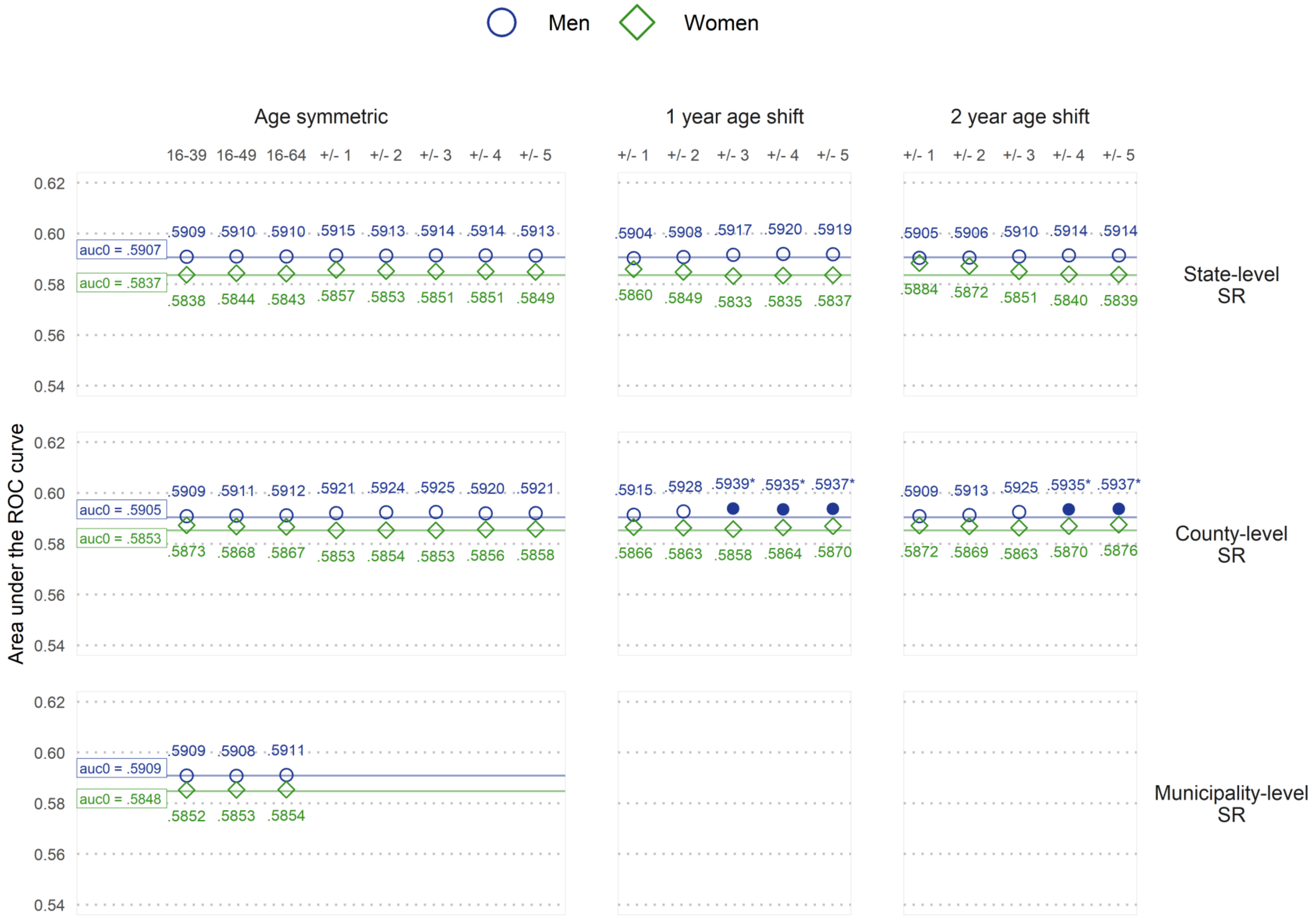

Area under the ROC curve based on multi-level logistic regressions predicting the probability to report a surplus of same-sex contacts. All models included random intercepts for individuals & administrative entites, and adjusted for age, age², education, east/west Germany, size of residential municipality, employment status, and parental status. Solid shapes & * indicate p<.05 for improved discrimination relative to an equivalent model without any sex ratio measure (acu0, horizontal line). Differences in auc0 result from diverging sample sizes due to missing population data (see Table 4)

Fig. S 2: Correlation coefficients for the association of local sex ratio measures with subjective surplus encounters with same-sex individuals

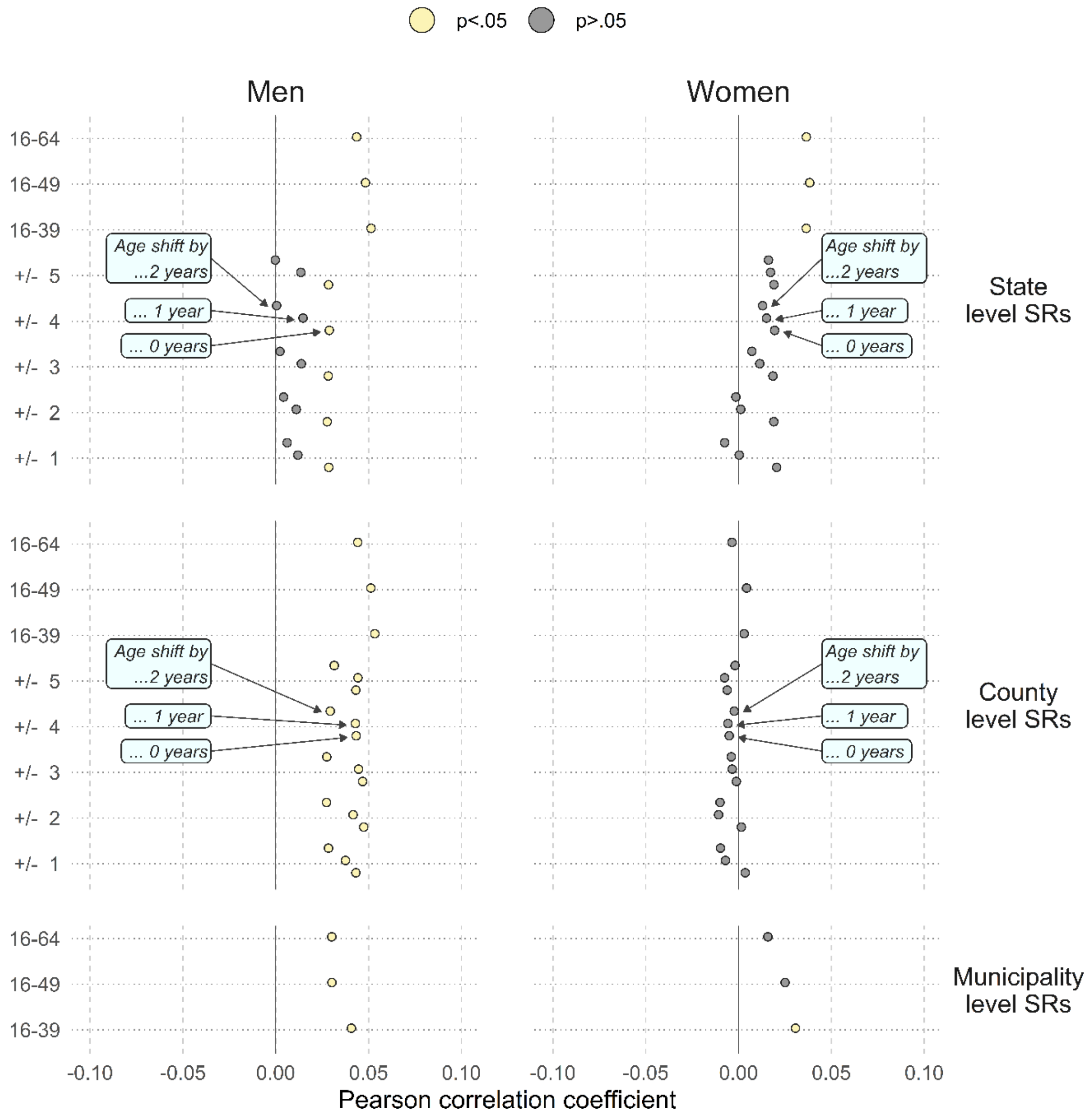

Correlation coefficients for the association between local sex ratio measures and the reported surplus encounters with same-sex individuals. For age-specific local sex ratios, three variants are displayed: age-symmetric (slightly below the respective horizontal grid line), age-shifted by one year (at the grid line) and age-shifted by two year (above the grid line). Age shifted sex ratios included male age cohorts are one or two years older than their female counterpart groups, respectively. Age symmetric sex ratios include men and women of the same age stages. Yellow-filled shapes indicate significant correlations ($p < .05$); grey-filled shapes indicate $p > .05$.

Fig. S 3: Standardised linear regression coefficients for the prediction of reported surpluses of encounters with same-sex individuals from state-level proportions of men

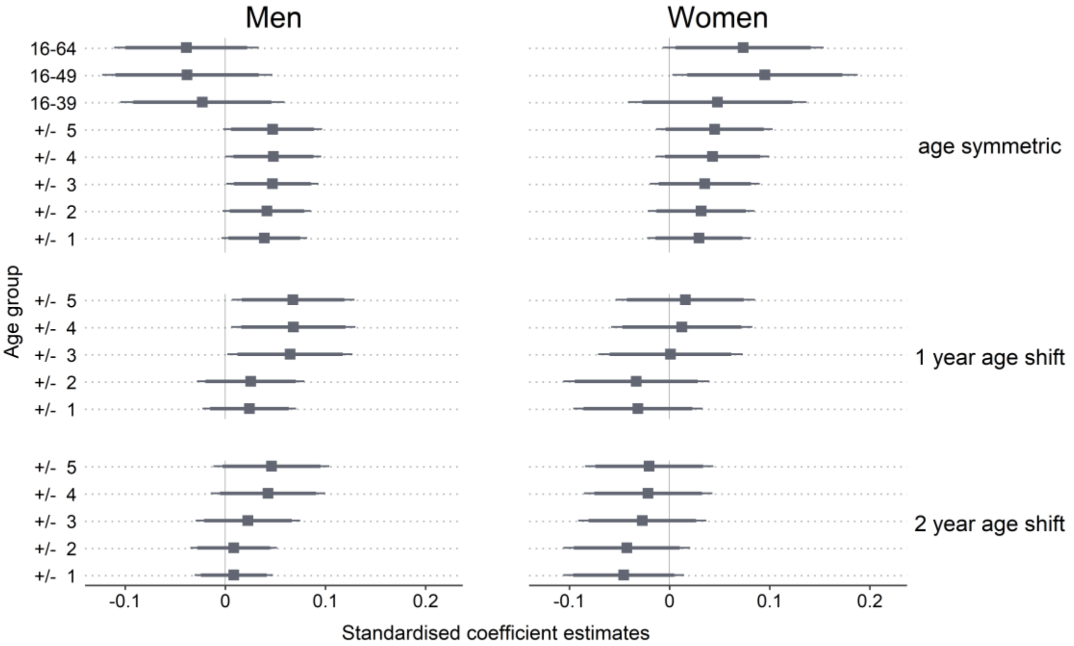

Standardised regression coefficients reflecting changes in the predicted score of surplus of same-sex encounters for a one standard deviation increase in the male population share.
Models included random intercepts for individuals & states and adjusted for age, age², education, east/west Germany, size of residential municipality, employment status, and parental status.
Error ranges indicate 90% confidence intervals (CIs; thick horizontal lines) and 95% CIs (thin horizontal lines).
N (men) = 8,817; N (female) = 6,029
ICC (men) = 0.0035; ICC (women) = 0.0037

Fig. S 4: Standardised linear regression coefficients for the prediction of reported surpluses of encounters with same-sex individuals from county-level proportions of men

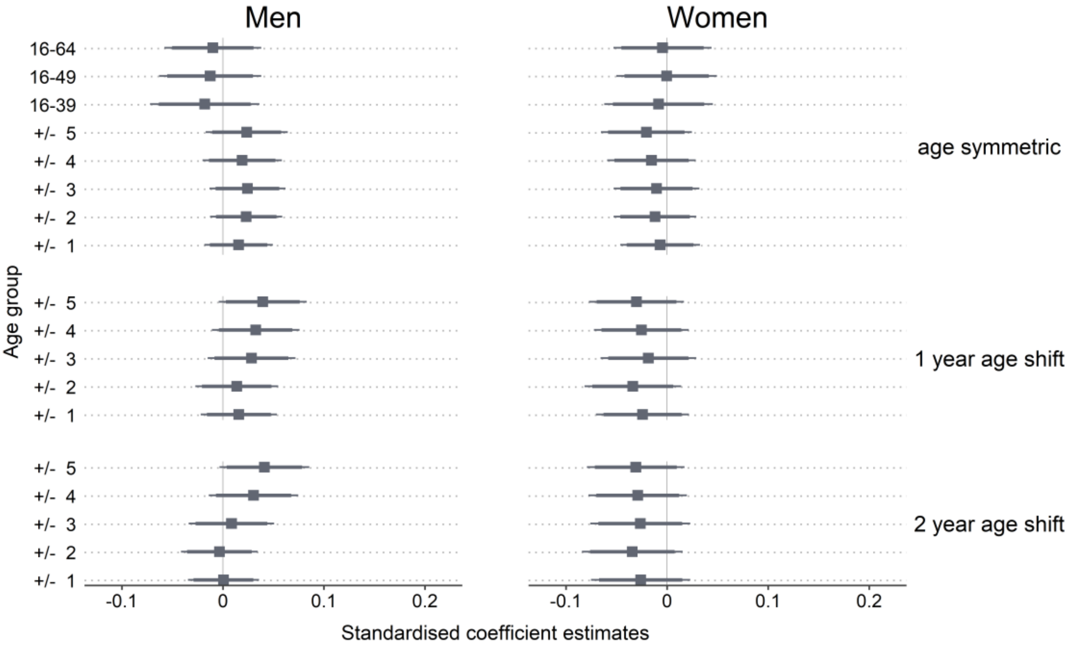

Standardised regression coefficients reflecting changes in the predicted score of surplus of same-sex encounters for a one standard deviation increase in the male population share.
Models included random intercepts for individuals & counties and adjusted for age, age², education, east/west Germany, size of residential municipality, employment status, and parental status.
Error ranges indicate 90% confidence intervals (CIs; thick horizontal lines) and 95% CIs (thin horizontal lines).
N (men) = 8,807; N (female) = 6,022
ICC (men) = 0.0311; ICC (women) = 0.0254

Fig. S 5: Standardised linear regression coefficients for the prediction of reported surpluses of encounters with same-sex individuals from municipality-level proportions of men

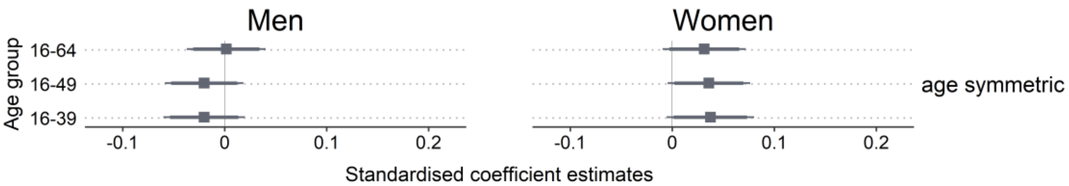

Standardised regression coefficients reflecting changes in the predicted score of surplus of same-sex encounters for a one standard deviation increase in the male population share.
Models included random intercepts for individuals & municipalities and adjusted for age, age², education, east/west Germany, size of residential municipality, employment status, and parental status.
Error ranges indicate 90% confidence intervals (CIs; thick horizontal lines) and 95% CIs (thin horizontal lines).
N (men) = 8,814; N (female) = 6,025
ICC (men) = 0.0365; ICC (women) = 0.0154

Fig. S 6: Average marginal effects from multinomial logistic regression models predicting reported surpluses of encounters with same-sex individuals from state-level proportions of men.

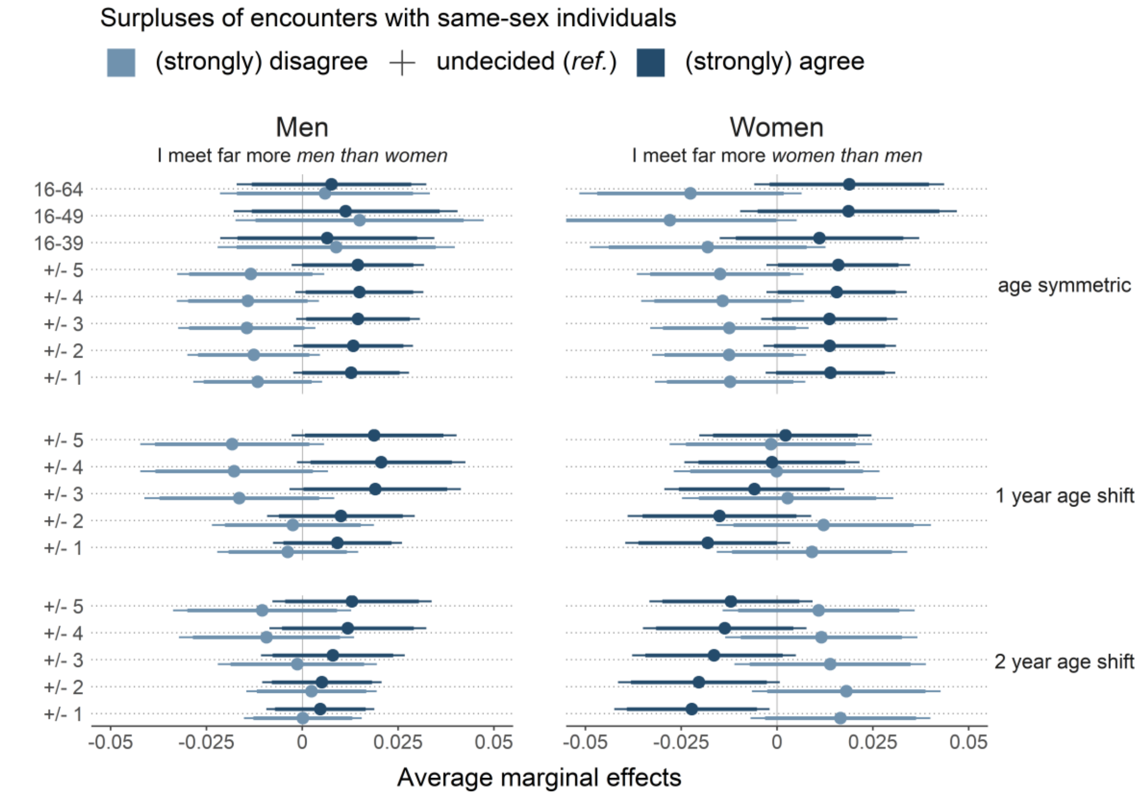

Average marginal effects reflecting changes in the probability to report a (strong) agreement (4|5) or disagreement (1|2) with the surplus of same-sex encounters indicator relative to the middle category (3) for a one standard deviation increase in the male population share.
Models included random intercepts for individuals & states and adjusted for age, age², education, east/west Germany, size of residential municipality, employment status, and parental status.
Error ranges indicate 90% confidence intervals (CIs; thick horizontal lines) and 95% CIs (thin horizontal lines).
N (men) = 8,817; N (female) = 6,029

Fig. S 7: Average marginal effects from multinomial logistic regression models predicting reported surpluses of encounters with same-sex individuals from county-level proportions of men.

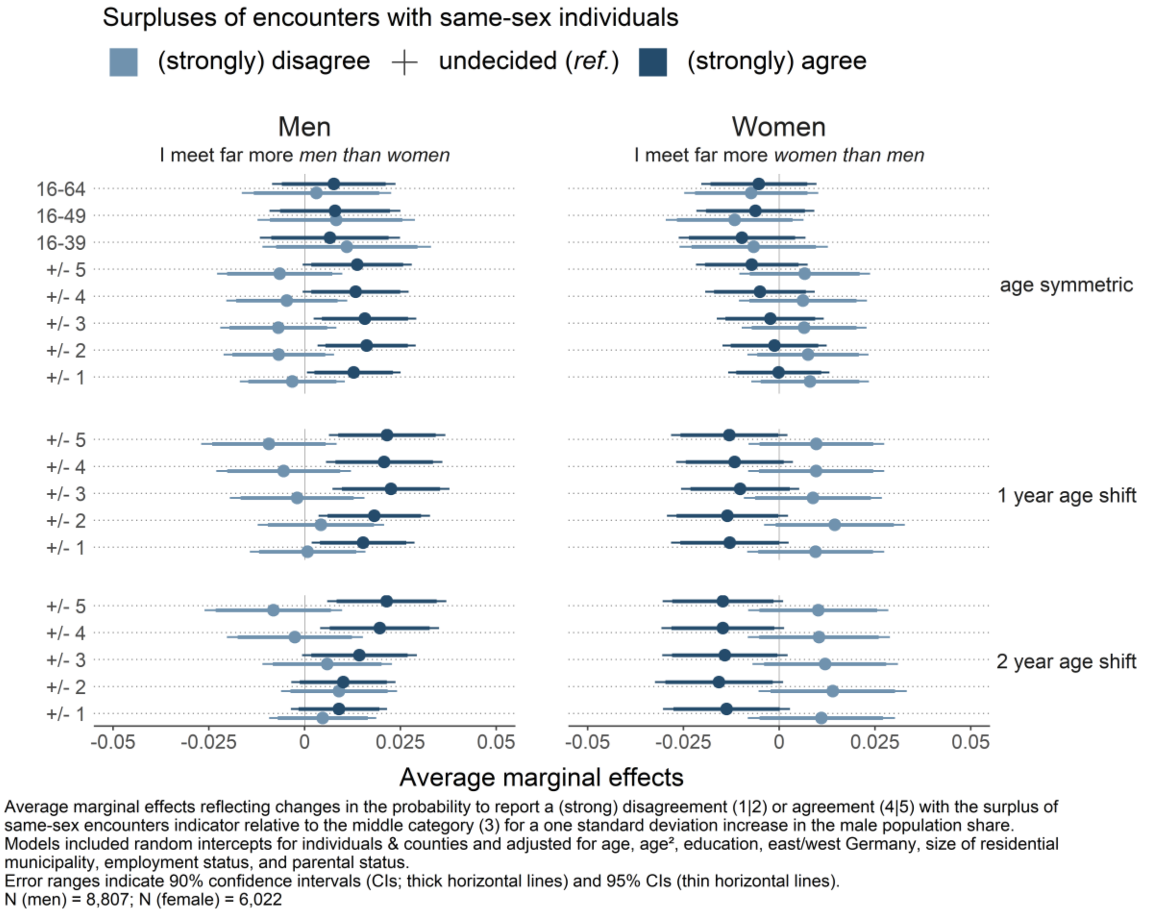

Fig. S 8: Average marginal effects from multinomial logistic regression models predicting reported surpluses of encounters with same-sex individuals from municipality-level proportions of men.

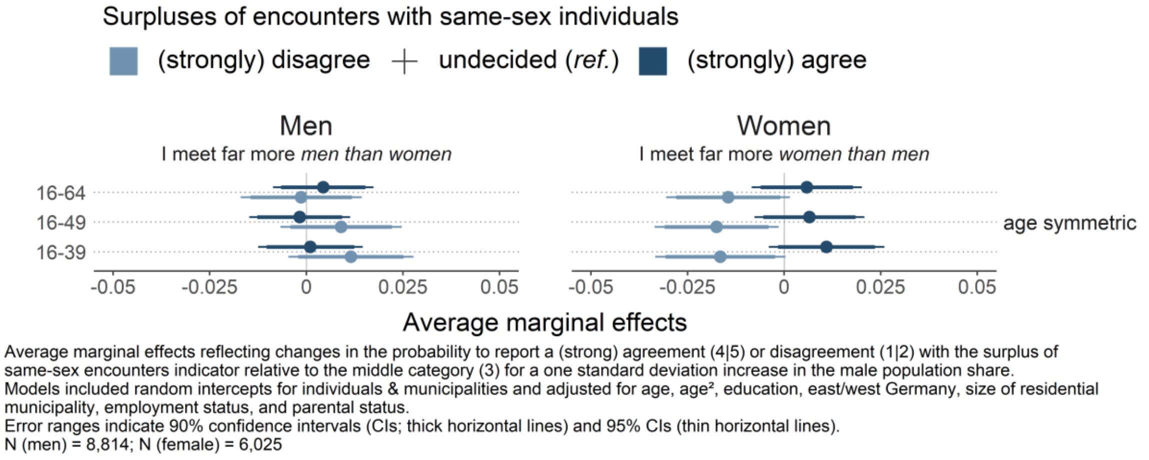

Table S1. Full logistic regression tables for the prediction of reported surpluses of encounters with same-sex individuals

		State level				County level				Municipality level			
		Men		Women		Men		Women		Men		Women	
		β	se	β	se	β	se	β	se	β	se	β	se
**Fixed Parts**													
Prop. men aged 16-39 (standardised)		0.0555	0.106	0.0645	0.121	0.0475	0.0661	-0.0689	0.072	0.0114	0.0493	0.102	0.0584
Age (standardised)	Age	0.376*	0.0661	0.304**	0.0944	0.364*	0.0652	0.306**	0.0933	0.363*	0.065	0.3**	0.0931
	Age²	-0.112***	0.0442	0.113	0.0659	-0.108***	0.0444	0.0918	0.0644	-0.108***	0.0443	0.0862	0.0643
Education	Basic/lower secondary	*ref.*		*ref.*		*ref.*		*ref.*		*ref.*		*ref.*	
	Higher secondary	-0.147	0.0985	0.324***	0.133	-0.152	0.0985	0.323***	0.133	-0.139	0.0984	0.327***	0.132
East/West	Western Germany	*ref.*		*ref.*		*ref.*		*ref.*		*ref.*		*ref.*	
	Former GDR	0.139	0.242	0.138	0.299	0.146	0.147	0.252	0.166	0.24***	0.117	0.103	0.142
Size of residential municipality	<5000	0.108	0.127	-0.122	0.173	0.0998	0.137	-0.0979	0.181	0.0663	0.136	-0.138	0.181
	5000-20000	*ref.*		*ref.*		*ref.*		*ref.*		*ref.*		*ref.*	
	20k-50k	0.337**	0.123	0.0324	0.156	0.298***	0.132	0.0535	0.163	0.301***	0.13	0.035	0.162
	50k-100k	-0.0356	0.17	0.22	0.211	-0.0964	0.181	0.22	0.221	-0.116	0.178	0.271	0.217
	100k-500k	0.143	0.135	0.0928	0.17	0.142	0.152	0.0724	0.186	0.0965	0.144	0.157	0.179
	>500k	-0.015	0.15	0.00175	0.179	0.0987	0.196	-0.161	0.217	0.00563	0.18	-0.00949	0.206
Employment status	In training	-0.343*	0.0913	0.0874	0.125	-0.344*	0.0917	0.103	0.125	-0.351*	0.0917	0.0965	0.125
	Non-working	0.0663	0.126	-0.172	0.162	0.08	0.126	-0.169	0.161	0.0886	0.127	-0.181	0.162
	Working	*ref.*		*ref.*		*ref.*		*ref.*		*ref.*		*ref.*	
Parental status	Not a parent	*ref.*		*ref.*		*ref.*		*ref.*		*ref.*		*ref.*	
	At least one child	-0.404***	0.192	-0.117	0.177	-0.417***	0.192	-0.111	0.176	-0.435***	0.191	-0.0916	0.175
	Constant	-1.054		-2.059		-1.065		-2.022		-1.065		-2.013	
**Random Parts**													
	τ²	0.004		0.043		0.129		0.138		0.085		0.125	
	σ²	2.617		2.925		2.518		2.786		2.562		2.802	
	AIC	9,912.15		6,488.39		9,914.35		6,500.48		9,938.36		6,517.99	
	BIC	10,025.50		6,595.66		10,027.69		6,607.73		10,051.70		6,625.25	
	LogLikelihood	-4,940.08		-3,228.19		-4,941.18		-3,234.24		-4,953.18		-3,242.99	
	N	8817		6029		8807		6022		8814		6025	

***p<0.001
**p<0.01
*p<0.05

Results for multilevel linear regression models for the dependent variable surplus of encounter with same-sex individuals. Coefficients are reported as logits. Models include individual-level variables and ASR, income and education as fixed effects and the respective entity as a random effect. The terms σ² and τ² represent the variance between administrative entities and individuals, respectively. The coefficients for individual-level control variables are virtually unchanged across models using different sex ratio specifications.

Table S2. Full regression tables for event history models of transition into relationships

		State level				County level				Municipality level			
		Men		Women		Men		Women		Men		Women	
		β	se	β	se	β	se	β	se	β	se	β	se
**Fixed Parts**													
Surplus of encounters with same-sex individuals		-0.261***	0.0723	-0.39***	0.0801	-0.26***	0.0727	-0.392***	0.0813	-0.26***	0.0727	-0.389***	0.0806
Prop. of men aged 16-39 (standardised)		0.0568	0.0924	-0.00117	0.0827	-0.0398	0.0515	0.0763	0.0531	-0.000429	0.0416	-0.0512	0.0407
Years at risk	1	*ref.*		*ref.*		*ref.*		*ref.*		*ref.*		*ref.*	
	2	-0.172*	0.0828	-0.148	0.0865	-0.168*	0.0832	-0.14	0.0874	-0.17*	0.0832	-0.148	0.087
	3	-0.338***	0.0936	-0.263**	0.101	-0.333***	0.094	-0.24*	0.102	-0.333***	0.0942	-0.257*	0.102
	4	-0.35**	0.108	-0.399**	0.122	-0.352**	0.108	-0.37**	0.123	-0.348**	0.108	-0.394**	0.123
	5	-0.753***	0.137	-0.77***	0.162	-0.748***	0.137	-0.747***	0.163	-0.747***	0.137	-0.766***	0.163
	6	-0.818***	0.186	-0.823***	0.217	-0.793***	0.187	-0.796***	0.22	-0.798***	0.187	-0.809***	0.219
Age (standardised)	Age	0.00871	0.0547	-0.268***	0.0683	0.0104	0.0551	-0.265***	0.0696	0.011	0.0553	-0.273***	0.069
	Age²	-0.161***	0.0415	-0.127*	0.054	-0.16***	0.0417	-0.131*	0.0549	-0.162***	0.0418	-0.126*	0.0545
Education	Basic/lower secondary	*ref.*		*ref.*		*ref.*		*ref.*		*ref.*		*ref.*	
	Higher secondary	0.183*	0.0865	0.12	0.0941	0.178*	0.0871	0.13	0.0956	0.181*	0.0872	0.13	0.0949
East/West	Western Germany	*ref.*		*ref.*		*ref.*		*ref.*		*ref.*		*ref.*	
	Former GDR	-0.116	0.21	0.129	0.2	0.0561	0.109	0.0441	0.117	0.00264	0.0903	0.183	0.0936
Size of residential municipality	<5000	0.0372	0.0971	0.108	0.112	0.0403	0.102	0.0559	0.122	0.0285	0.103	0.119	0.116
	5000-20000	*ref.*		*ref.*		*ref.*		*ref.*		*ref.*		*ref.*	
	20k-50k	0.144	0.0922	-0.0123	0.1	0.145	0.0959	-0.0352	0.109	0.142	0.0973	-0.0192	0.104
	50k-100k	0.236	0.122	0.0285	0.136	0.24	0.127	0.0114	0.147	0.23	0.129	-0.001	0.14
	100k-500k	0.0134	0.102	-0.0524	0.112	-0.0126	0.111	-0.0367	0.127	0.00458	0.11	-0.0815	0.118
	>500k	0.0689	0.115	-0.147	0.115	0.0166	0.133	-0.147	0.149	0.0459	0.132	-0.21	0.132
Employment status	In training	-0.0813	0.0828	-0.333***	0.0955	-0.0805	0.0834	-0.327***	0.0967	-0.0794	0.0834	-0.333***	0.0961
	Non-working	-0.719***	0.149	-0.17	0.132	-0.71***	0.15	-0.162	0.134	-0.712***	0.15	-0.16	0.133
	Working	*ref.*		*ref.*		*ref.*		*ref.*		*ref.*		*ref.*	
Parental status	Not a parent	*ref.*		*ref.*		*ref.*		*ref.*		*ref.*		*ref.*	
	At least one child	0.707***	0.149	0.315*	0.128	0.712***	0.151	0.286*	0.131	0.718***	0.151	0.318*	0.129
	Constant	-1.117		-0.565		-1.16		-0.557		-1.146		-0.585	

	State level				County level				Municipality level			
	Men		Women		Men		Women		Men		Women	
	β	se	β	se	β	se	β	se	β	se	β	se
**Random Part**												
σ²	0.0000		0.0045		0.0245		0.0650		0.0326		0.0350	
AIC	6397.00		5230.90		6391.70		5215.40		6397.50		5227.70	
BIC	6533.10		5365.70		6534.60		5350.20		6540.40		5362.50	
LogLikelihood	-3178.50		-2594.50		-3174.90		-2586.70		-3177.80		-2592.90	
N	6655		4532		6647		4525		6653		4530	

***p<0.001
**p<0.01
*p<0.05

Results for multilevel linear logistic regression models for the dependent variable transition into relationships. Coefficients are reported as logits. The term σ² represents the variance between the respective administrative entities. The coefficients for individual-level control variables are virtually unchanged across models using different sex ratio specifications.

Fig. S 9: Average marginal effects of surpluses of encounters with same-sex individuals (SESSI) and proportions of men on union formation from combined and separate models

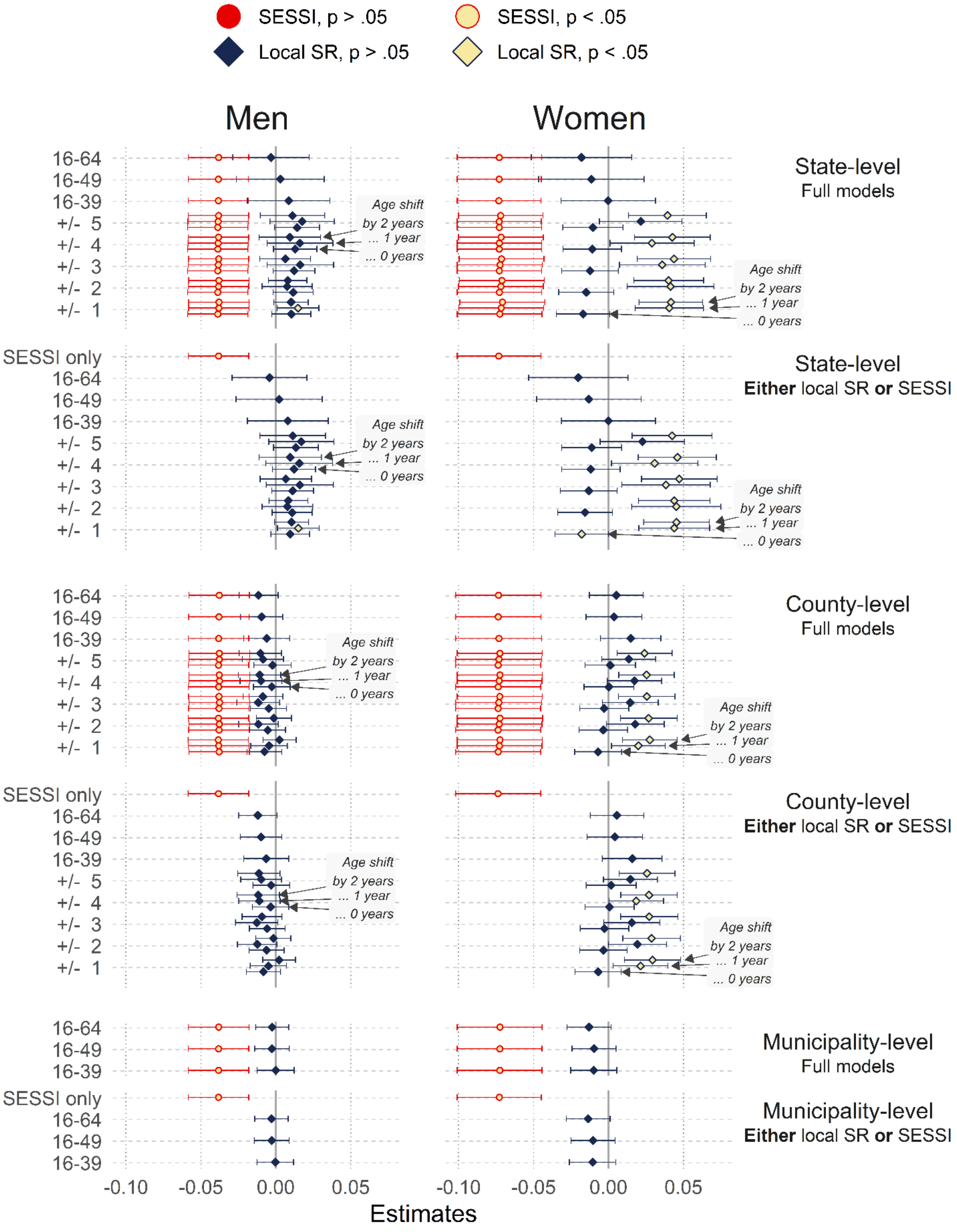

Average marginal effects from logistic discrete-time event history models predicting relationship formation.
Coefficients for local proportions of men reflect changes in the probability to enter a relationship for a one standard deviation increase in the male population share.
Coefficients for surplus encounters with same-sex individuals reflect changes in the probability to enter a relationship when reporting a surplus of encounters with individuals of one's own sex, compared to undecided or negative answers.
Models included random intercepts for individuals & administrative entities. All models and adjusted for age, age², education, east/west Germany, size of residential municipality, employment status, and parental status.
For age-specific local sex ratios, three variants are displayed: age-symmetric (slightly below the respective horizontal grid line), age-shifted by one year (at the grid line) and age-shifted by two year (above the grid line). Age shifted sex ratios include the number of men who are one and two years older than their female counterpart groups to incorporate age hypergamy. Age symmetric sex ratios include men and women of the same age brackets. Yellow-filled shapes indiciate p<.05
